# A review of the *Eviotazebrina* complex, with descriptions of four new species (Teleostei, Gobiidae)

**DOI:** 10.3897/zookeys.1057.66675

**Published:** 2021-08-27

**Authors:** Luke Tornabene, David W. Greenfield, Mark V. Erdmann

**Affiliations:** 1 School of Aquatic and Fishery Sciences, and the Burke Museum of Natural History and Culture, University of Washington, 1122 NE Boat Street, Seattle, Washington, 98105, USA; 2 Research Associate, Department of Ichthyology, California Academy of Sciences, 55 Music Concourse Dr., Golden Gate Park, San Francisco, California 94118-4503, USA; 3 Professor Emeritus, University of Hawaii. Mailing address: 944 Egan Ave., Pacific Grove, CA 93950, USA; 4 Conservation International Aotearoa, University of Auckland, 23 Symonds Street, Auckland 1010, New Zealand; 5 Research Associate, Department of Ichthyology, California Academy of Sciences, 55 Music Concourse Dr., Golden Gate Park, San Francisco, CA 94118-4599, USA

**Keywords:** coral-reef fishes, dwarfgoby, *
Eviota cometa
*, gobies, ichthyology, taxonomy, systematics

## Abstract

The *Eviotazebrina* complex includes eight species of closely-related dwarfgobies, four of which are herein described as new. The complex is named for *Eviotazebrina* Lachner & Karnella, 1978, an Indian Ocean species with the holotype from the Seychelles Islands and also known from the Maldives, which was once thought to range into the Gulf of Aqaba and the Red Sea eastward to the Great Barrier Reef of Australia. Our analysis supports the recognition of four genetically distinct, geographically non-overlapping, species within what was previously called *E.zebrina*, with *E.zebrina* being restricted to the Indian Ocean, *E.marerubrum***sp. nov.** described from the Red Sea, *E.longirostris***sp. nov.** described from western New Guinea, and *E.pseudozebrina***sp. nov.** described from Fiji. The caudal fin of all four of these species is crossed by oblique black bars in preservative, but these black bars are absent from the four other species included in the complex. Two of the other species within the complex, *E.tetha* and *E.gunawanae* are morphologically similar to each other in having the AITO cephalic-sensory pore positioned far forward and opening anteriorly. *Eviotatetha* is known from lagoonal environments in Cenderawasih Bay and Raja Ampat, West Papua, and *E.gunawanae* is known only from deeper reefs (35–60 m) from Fakfak Regency, West Papua. The final two species are *E.cometa* which is known from Fiji and Tonga and possesses red bars crossing the caudal fin (but lost in preservative) and a 9/8 dorsal/anal-fin formula, and *E.oculineata***sp. nov.**, which is described as new from New Guinea and the Solomon Islands, and possesses an 8/7 dorsal/anal-fin formula and lacks red caudal bars. *Eviotaoculineata* has been confused with *E.cometa* in the past.

## Introduction

**Figure 1.**
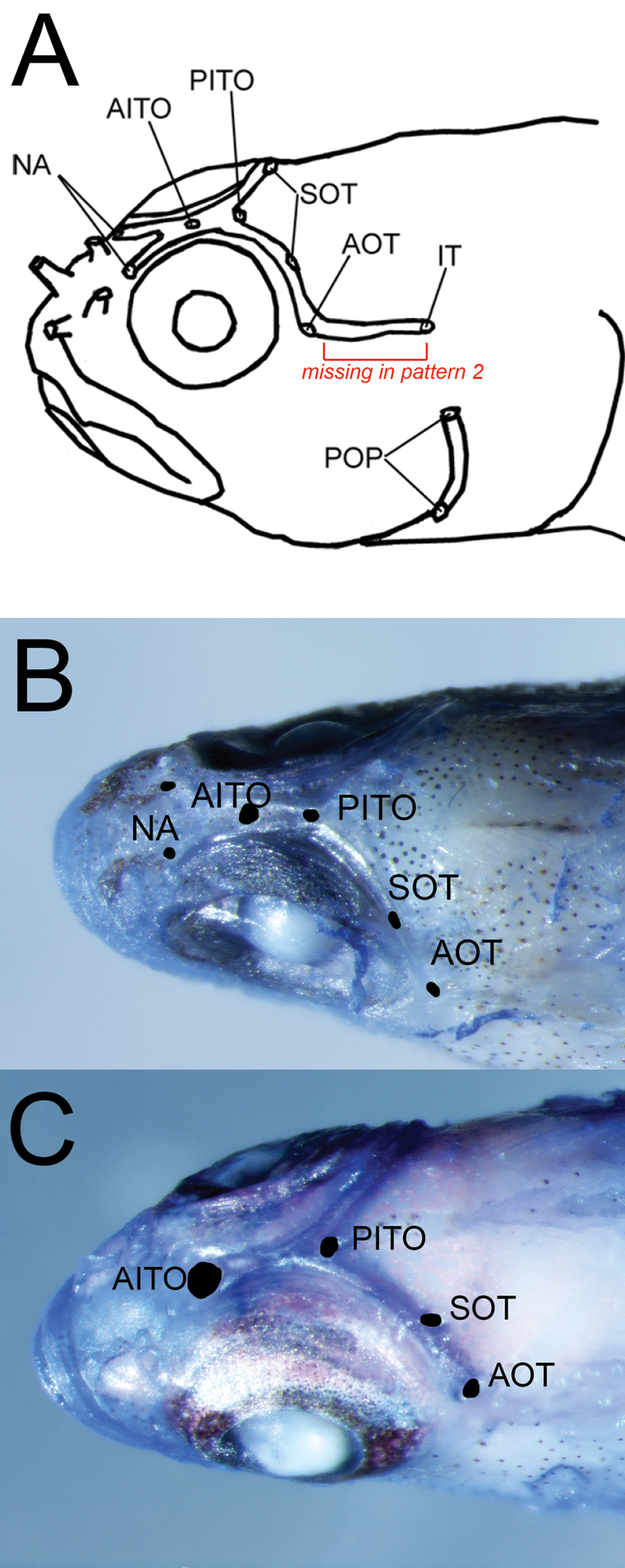
Head pore patterns relevant to the *Eviotazebrina* complex **A** complete pore pattern (pattern 1 of Lachner and Karnella 1980). Note that the IT pore and associated section of the lateralis canal are absent in pore pattern 2 and in all species of the *E.zebrina* complex. Figure modified from Tornabene et al. (2013)**B** pattern 2, found in all species of the *E.zebrina* complex except *E.tetha* and *E.gunawanae*. Photograph of CAS 246248, *E.longirostris***C** pattern found in *E.tetha* and *E.gunawanae*. Photograph of CAS 246247, *E.tetha*. Note the absence of NA pores and the anterior-facing AITO pore. Pore abbreviations follow Lachner and Karnell (1980).

The genus *Eviota*, commonly known as dwarfgobies, contains 124 species (Greenfield 2021; Greenfield and Erdmann 2021), making it one of the most speciose genera of marine fishes. Several putatively widespread species of *Eviota* displaying morphological variation have recently been shown to be complexes of distinct species that are often distinguishable by a combination of genetic differences, subtle morphological characters, and/or differences in live coloration (Greenfield and Tornabene 2014; Tornabene et al. 2015, 2016). Another example of this phenomenon is demonstrated by the *Eviotazebrina* complex, a group containing eight genetic lineages based on mtDNA, including the nominal species *Eviotazebrina* Lachner & Karnella, 1978, *E.cometa* Jewett & Lachner, 1983, *E.gunawanae*Greenfield et al., 2019, *E.tetha* Greenfield & Erdmann, 2014, and several undescribed species (Greenfield et al. 2019). All species in this complex possess: (i) unbranched pectoral-fin rays; (ii) reduced fifth pelvic-fin rays (absent or rudimentary to ~ 15% length of fourth ray); (iii) pore patterns lacking only the IT pores, or both the IT and nasal pores Figure 1); (iv) fourth pelvic-fin ray with only 3–7 branches; (v) a red or dark brown lateral stripe on the body (stripe faint in *E.pseudozebrina* sp. nov.) that terminates in distinct black spot on the base of the caudal fin that is sometimes bordered with yellow in life (spot faint in *E.tetha*). The combination of the lateral stripe and black caudal spot are present only in two other species, *E.sebreei* Jordan & Seale, 1906 and *E.punyit*Tornabene et al., 2016, both of which differ from the *E.zebrina* complex in having a pore pattern lacking the PITO pore, and in having a fourth pelvic-fin ray with many more branches (11–15 vs. 3–7).

**Figure 2.**
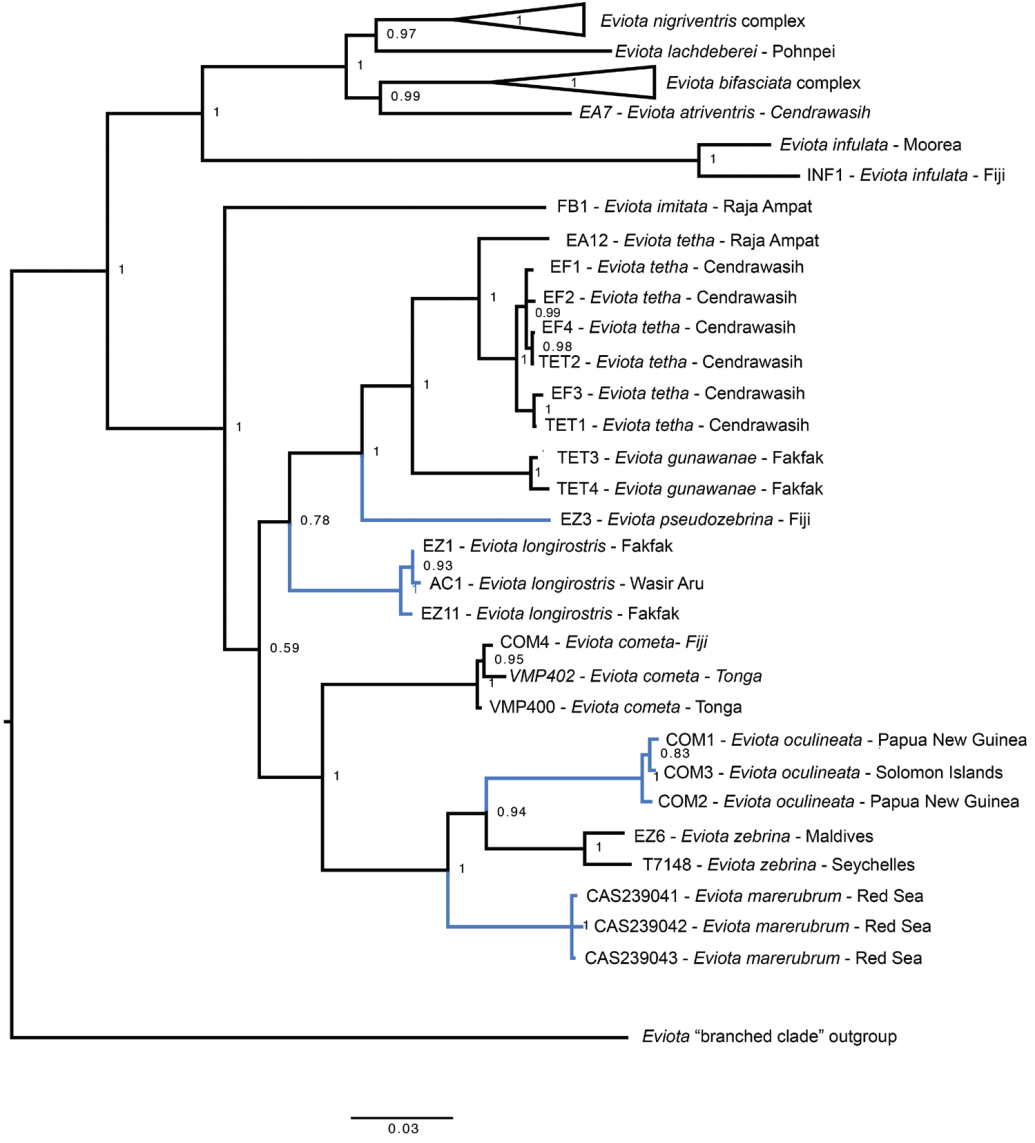
Molecular phylogeny based on COI and Ptr sequences. Branch lengths are substitutions/site, node labels are Bayesian posterior probabilities. Blue branches indicate new species described here. Labels before species name refer to tissue numbers.

*Eviotazebrina* was described based on type material from the Seychelles Islands, with non-type specimens from the Red Sea and other Indian Ocean localities east to the Great Barrier Reef, Australia. Lachner and Karnella (1978) noted geographic variation in meristic characters and pigmentation patterns among populations but refrained from splitting the groups into named species. During their survey of the marine fishes of Fiji (1999–2003), Greenfield and Randall identified one of the dwarfgobies collected as *Eviotazebrina*, the first record of that species from Oceania at that time (Randall 2005). When Herler and Hilgers (2005) published color photographs of *E.zebrina* from the Red Sea that differed from the color of specimens from Fiji, it raised suspicions that the specimens from Fiji might be undescribed. In 2017 MVE collected specimens and DNA tissue of *E.zebrina* from the southern Lau Islands, Fiji, and in 2018 specimens and tissue of *E.zebrina* from the Maldives, an Indian Ocean location. Greenfield et al. (2019) included DNA sequences of *E.zebrina* from the Red Sea, the Maldives, Seychelles Islands, western New Guinea, and Fiji in a molecular phylogeny of this complex (Figure 2), spanning most of the known range of *E.zebrina*. These data showed that *E.zebrina* indeed represented a species complex, with specimens of *Eviota “zebrina*” from the Red Sea, western New Guinea, and Fiji all representing lineages that are distinct from those in the Indian Ocean. We herein describe the specimens from Fiji as *Eviotapseudozebrina* sp. nov., those from western New Guinea as *E.longirostris* sp. nov., and the Red Sea specimens as *E.marerubrum* sp. nov.

*Eviotacometa* is also a member of the *E.zebrina* complex, and based on the phylogenetic tree of Greenfield et al. (2019), there are two lineages putatively identified as *E.cometa*. *Eviotacometa* was described by Jewett and Lachner (1983) from specimens in Fiji (type locality) and many non-type specimens across the western and central Pacific Ocean. As was the case with *E.zebrina*, the authors noted that *E.cometa* displayed some morphological variation across its geographic range but refrained from recognizing morphotypes as distinct species. Specifically, Jewett and Lachner (1983) noted that there were two distinct counts in the dorsal- and anal-fin rays, 9/8 (as in the holotype) vs 8/7, but in some locations, including the type locality in Fiji, both counts were present, leading to the conclusion that this was intraspecific variation. No differences in live coloration were noted at the time of description. Recent live photographs of specimens of *E.cometa* have revealed the presence of two distinct color morphs, both of which occur near the type locality of Fiji, and correspond to the genetic lineages shown by Greenfield et al. (2019). We show here that these genetically distinct color morphs correspond to the groups of possessing two different fin-ray counts noted by Jewett and Lachner (1983). We describe the new species here as *E.oculineata* sp. nov. Lastly, we provide a detailed comparison of the eight known species in this complex and a taxonomic key to aid in their identification.

The addition of these four new species raises the total number of species in the genus to 127.

## Materials and methods

Counts and measurements, descriptions of fin morphology and the cephalic sensory-canal pore patterns follow Lachner and Karnella (1980) and Jewett and Lachner (1983). Dorsal/anal fin-ray formula counts (e.g., 9/8) only include segmented rays. For counts of rays in dorsal, anal, and pectoral fins in the Description sections below, we list the holotype first followed by the range of counts for the entire type series, with the frequency of that count in square brackets.

Measurements were made to the nearest 0.1 mm using an ocular micrometer or dial calipers, and are presented as percentage of Standard Length (**SL**). Measurements of the holotype are listed first, followed by the range and average for all measured specimens of the type series in parenthesis. Lengths are given as standard length (SL), measured from the median anterior point of the upper lip to the base of the caudal fin (posterior end of the hypural plate); origin of the first dorsal fin is measured from the median anterior point of the upper lip to the anterior base of the first dorsal-fin spine; origin of the second dorsal fin is measured from the median anterior point of the upper lip to the anterior base of its spine; origin of the anal fin is measured from the median anterior point of the upper lip to the anterior base of its spine; body depth is measured at the center of the first dorsal fin; head length is taken from the upper lip to the posterior end of the opercular membrane; orbit diameter is the greatest fleshy diameter; snout length is measured from the median anterior point of the upper lip to the nearest fleshy edge of the orbit; upper jaw length is the distance from the anterior tip of the premaxilla to the end of the upper margin of the dentary where the maxilla joins; caudal-peduncle depth is the least depth, and caudal-peduncle length the horizontal distance between verticals at the rear base of the anal fin and the caudal-fin base; pelvic-fin length is measured from the base of the pelvic-fin spine to the tip of the longest pelvic-fin soft ray. Cyanine Blue 5R (acid blue 113) stain and an air jet were used to make the cephalic sensory-canal pores more obvious (Akihito et al. 1993, 2002; Saruwatari et al. 1997). A digital radiograph was taken of the holotype of *Eviotacometa* to confirm counts of dorsal- and anal-fin rays.

Sequences for the new species and additional specimens from the *E.zebrina* complex were sequenced in Greenfield et al. (2019) and our prior studies on *Eviota* (Tornabene et al. 2013, 2015, 2016; Greenfield et al. 2019). For those studies, we sequenced a segment of the mitochondrial gene *cytochrome c oxidase subunit I* (COI) using the primers GobyL6468 and GobyH7696 (Thacker 2003) or FishF-1 and FishR-1 (Ward et al. 2005), and the nuclear gene *Protease III* (Ptr) using the primers PtrF2 and PtrR2 (Yamada et al. 2009). The PCR conditions follow that of Tornabene et al. (2016). Additional sequences that were putatively identified as *E.zebrina* were added from BOLD or GenBank. Sequences were aligned in Geneious v.6.0.6 (Biomatters; www.geneious.com). The final alignment consisted of 1173 bp of COI, and 614 bp of Ptr. A phylogenetic analysis of the concatenated alignment was done using Bayesian Inference in the software MrBayes v.3.2 (Ronquist et al. 2012), partitioning by gene. Substitution models were chosen using PartitionFinder2 (Lanfear et al. 2016). The analysis was run for 10^6^ generations, discarding the first 10% of trees as burn-in.

## Taxonomic account

### Eviota pseudozebrina

Taxon classificationAnimaliaPerciformesGobiidae

Tornabene, Greenfield & Erdmann, 2021

AF161F93-1F99-538E-9BC7-DADC3E271BE6

**Figure 3.**
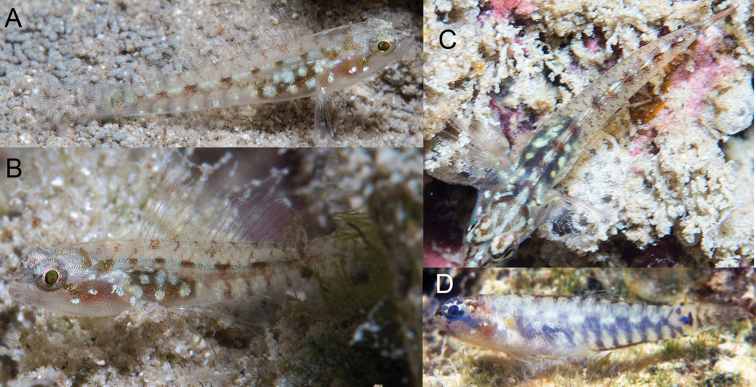
*Eviotapseudozebrina*, live. **A–C** CAS 244078, 10.4 mm SL male, Fiji **D** Underwater photograph of fresh specimen from Fiji, reproduced with permission from Randall (2005).

**Figure 4.**
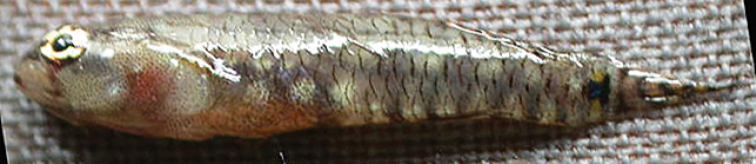
*Eviotapseudozebrina*, fresh specimen, type series, CAS 246310.

**Figure 5.**
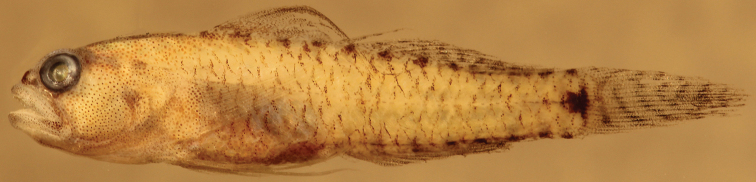
*Eviotapseudozebrina*, preserved holotype, CAS 228614, 16.6 mm SL male, Fiji.

Fijian zebra Dwarfgoby Figures 3 , 4 , 5

Eviota cf. zebrina : Greenfield et al. 2019: 64, fig. 9 (Fiji).

#### Material.

***Holotype*.** CAS 228614, 16.6 mm SL male, Fiji, N. Lau Group, Vanua Balavu Id., Bay of Islands, cove in Bay, 17°10.679'S, 179°01.558'W, 0–2.4 m, rock with green algae, rotenone, field number G03-40, 13 January 2003, D.W. Greenfield, R. Langston, & K. Longenecker. ***Paratypes*.** CAS 246310, 10 males, 14.0–18.3 mm SL, 11 females, 11.6–14.8 mm SL, taken with holotype. CAS 244078, male, 10.4 mm SL, Fiji, S. Lau Group, Matuku Lagoon, 19°09.115'S, 179°44.732'E, 3–5 m, clove oil & hand net, field number MVE-17-016, 14 May 2017, M.V. Erdmann. CAS 246250, 10.5 mm SL, Fiji, S. Lau Group, Matuku Lagoon, 19°09.115'S, 179°44.732'E, 3–5 m, tissue number EZ3, clove oil & hand net, field number MVE-17-016, 14 May 2017, M.V. Erdmann.

#### Non-type material.

All from Fiji - CAS 219786 (3), Viti Levu Id., off Suva; 228677 (8), Vanua Balavu Id., Bay of Islands; 228731 (1), Vanau Balavu Id., Bay of Islands; 228732 (2), Vanua Balavu Id., Balavu Harbor; 228743 (1), Viti Levu Id., Vatunisogasoga Reef; 228744 (1), Mago Id., Lau Group; 229544 (1), Viti Levu Id., E. of Nananu Passage; 229568 (2), Yadua Id., Talai Harbor; 229580 (14), Yadua Id., Tali Harbor; 229608 (15), Viti Levu Id., Nananui-i-cake Id.

#### Diagnosis.

A species of *Eviota* with a cephalic sensory-canal pore pattern lacking only the IT pore, pectoral-fin rays not branched, dorsal/anal-fin ray formula 9/8, 5^th^ pelvic-fin ray 6–16% of 4^th^ ray; dark rectangular to round spot on area of preural centrum followed by a dark vertical line over end of hypural plate; caudal fin crossed by six or seven dark vertical bars in preservation, naris long and black, body deep, 22–26% SL; usually 15 pectoral-fin rays. Color of body a translucent gray background and markings of white, brown, or black, with no red coloration.

#### Description.

Dorsal-fin elements VI+I,9, first dorsal triangular in shape, first three spines filamentous, 2^nd^ or 3^rd^ longest, reaching to 6^th^ soft ray of second dorsal fin in holotype when adpressed, all second dorsal-fin soft rays branched, last ray branched to base; anal-fin elements I,8 (7[1],8[9])), all soft rays branched, last ray branched to base; pectoral-fin rays 16 (14[1], 15 [11], 16 [5]), all unbranched, pointed, reaching to below second dorsal fin; 5^th^ pelvic-fin ray ~ 16% (6–16) of length of 4^th^ pelvic-fin ray; 4^th^ ray with 7 (3–7) branches, four segments between consecutive branches of 4^th^ pelvic-fin ray, pelvic-fin membrane reduced, no basal membrane; caudal fin with 11 branched and 17 segmented rays; lateral-line scales 24 (24[8], 25[2]); transverse scale rows 7; urogenital papilla of male smooth, long and narrow, expanded into a lateral horn at tip, extending past anal-fin spine; female papilla smooth, bulbous, with short finger-like projections on end; front of head rounded with an angle of ~ 60° from horizontal axis; mouth slanted obliquely upwards, forming an angle of ~ 60° to horizontal axis of body, lower jaw not projecting; maxilla extending posteriorly to rear of pupil; anterior tubular nares long, black, extending to center of upper lip; gill opening extending forward to below posteroventral edge of preoperculum; cephalic sensory-pore system missing only IT pore, cutaneous sensory papilla system similar to papilla pattern B-1 (of Lachner and Karnella 1980).

#### Measurements.

In percent SL, value of holotype followed by range and mean of holotype and nine other paratypes in parentheses. Head length 28 (27–32, 29); origin of first dorsal fin 36 (33–37, 35); origin of second dorsal fin 57 (54–60, 57); origin of anal fin 59.9 (56–62, 59); caudal-peduncle length 24 (23–30, 25); caudal-peduncle depth 12 (10–13, 12); body depth 23 (23–26, 23); eye diameter 9 (8–10, 9); snout length 5 (4–5, 4); upper-jaw length 11 (females 9–11, 10, males 10–12, 11); pectoral-fin length 39 (31–39, 33); greatest pelvic-fin length 33 (27–36, 32).

#### Color in life.

(Figure 3) Background color of head and body translucent gray. Body with eight yellow-white spots along vertebral column, separated by black areas. A faint, dusky brown lateral stripe positioned below vertebral column, starting over abdomen and continuing onto caudal peduncle. Abdomen dark brown to black with seven large, irregularly shaped bright white spots spaced over dark area. Ventral portion of flank with six short, faint, vertical brown bars connected to dusky lateral stripe, each bar separated from one another by a small, bright yellow-white iridescent spot. Dorsal midline with approximately 11–13 brown spots, starting on predorsal area and ending over caudal-fin base, spots formed from coalescing dark anterior scale margins. Caudal-fin base with group of large melanophores in advance of posterior end of hypural plate, followed by a dark brown vertical line over end of plate. Top of head with long white line in center extending from between eyes back to nape, a curved white line on each side of line, curved out and back to nape. A line of faint melanophores extending from 7 o’clock position of eye down across cheek. Reddish color of gill filaments shows through cheek, with two white spots at end of dark stripe from eye. Dark stripe extending onto snout from 3 o’clock position of eye, with another line medial to it, the two lines joining anteriorly to meet black tubular nares, diverging posteriorly to form a Y shape. Pupil of eye black with iridescent greenish-yellow hue, upper half of eye surface cream colored with scattered melanophores, lower half darker. Pectoral-fin base with single white spot and another on base of fin rays. The dark bars crossing the caudal fin in freshly dead specimens not as visible in life.

#### Color in freshly dead specimen.

(Figure 4). Generally pigmented as described for live specimens, but with the lateral stripe and ventral vertical bars on flank considerably darker than in life. Dark bars on caudal fin much darker in preservation than in life. A distinct vertical bar of melanophores extending ventrally from eye (positioned at ~ 7 o’clock) extending over cheek and jaws. Vertical bar on cheek present in life, but much lighter. Anterior scale margins over entire body with dark pigment, versus only some dorsal scales pigmented in live specimens.

#### Color in preservative.

(Figure 5). Background color of head and body light cream. Scale anterior margins lined with melanophores, making scale pattern obvious. Side of head and jaws, pectoral-fin base and nape peppered with large melanophores. Tubular nares entirely black. Dark cluster of melanophores on lower jaw at rictus. Dark blotch at center of preoperculum at level of bottom of eye, another above it at level of pupil and dark blotch behind upper half of eye. Pupil of eye gray, iris black. Lower surface of abdomen dark brown. Series of dark brown spots extending from front of first dorsal fin back along fin bases to caudal-fin base. Similar series of dark spots along anal-fin base and onto caudal peduncle. Caudal-fin base with distinct rectangular-shaped dark brown spot in advance of posterior end of hypural plate, followed by a dark brown vertical line over end of plate. Caudal fin peppered with melanophores and crossed by seven dark brown bars. Filamentous spines of first dorsal fin dark brown, remainder of fin heavily peppered with melanophores. Second dorsal fin peppered with melanophores and crossed by 4 dark brown bars. Anal fin peppered with melanophores. Pectoral and pelvic fins lightly peppered with melanophores.

#### Etymology.

The specific epithet is an adjective combining the Greek *pseudos* (lie) and *zebrina* (New Latin meaning zebra-marked), referring to its similarity to *Eviotazebrina*.

#### Distribution and habitat.

Definitively known only from Fiji, but specimens identified as *E.zebrina* are known from Wallis & Futuna and Tonga in Oceania; genetic analysis of specimens from these areas is required to verify if they in fact represent *E.pseudozebrina*. In our CAS collections, the largest samples were from habitats with rock and green algae (Greenfield and Randall 2016). Individuals collected by MVE were similarly from a lagoonal habitat with mixed sand, coral rubble, macroalgae and scattered live coral; in all cases specimens were collected from the intertidal to a maximum depth of 5 m.

### Eviota longirostris

Taxon classificationAnimaliaPerciformesGobiidae

Tornabene, Greenfield & Erdmann, 2021

026F2211-9F97-5B02-9EE3-30E187630A26

**Figure 6.**
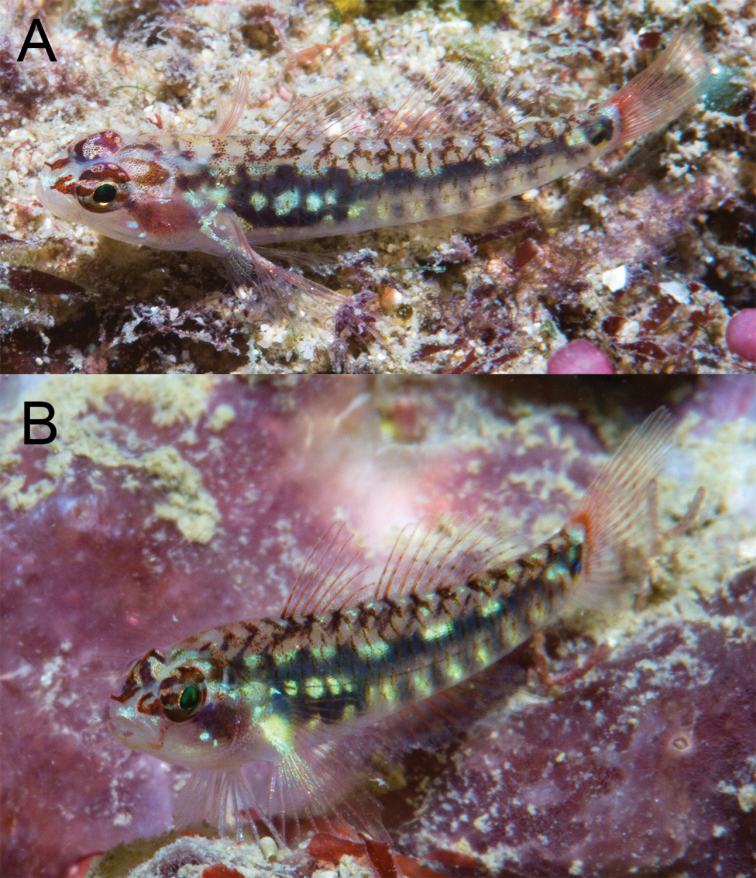
Underwater photograph of *Eviotalongirostris***A** paratype, CAS 246538, near type locality, West Papua, MVE-18-018 **B** specimen from type locality, West Papua, MVE-18-014.

**Figure 7.**
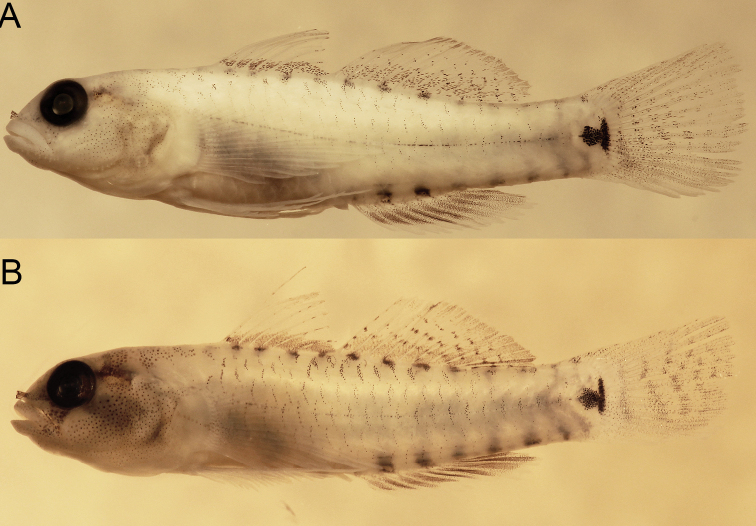
**A***Eviotalongirostris*, preserved holotype, MZB 26096 **B** paratype, CAS 246538, 15.0 mm SL male.

Long-snout Dwarfgoby Figures 6 , 7

Eviota cf. zebrina . Greenfield et al. 2019: 64, fig. 9 (West Papua).

#### Material.

***Holotype*.** MZB 26096, 17.6 mm SL female, Sariga, Kokas, Fakfak, West Papua, 02°36.367'S, 132°24.746'E, 5 m, clove oil & hand net, field number MVE-18-014, 8 March 2018, M.V. Erdmann. ***Paratypes*.** CAS 2246249, 5, taken with holotype. CAS 246249, 13.2 mm SL male, taken with holotype, tissue numbers EZ11-EZ15, preserved in 95% ethanol. CAS 246538, 15.0 mm SL male, 15.2 mm SL female, 02°38.527'S, 132°31.363'E, Fuum, Kokas, Fakfak, West Papua, 3 m, clove oil & hand net, field number MVE-18-018, 10 March 2018, M.V. Erdmann. CAS 246779 17.8 mm SL male, 03°54.784'S, 134°07.333'E, Bo’s Rainbow, Kaimana, West Papua, 2 m, clove oil & had net, field number MVE 19-026, 21 April 2019, M.V. Erdmann. CAS 246248, 2, tissue numbers EZ1 and EZ2, preserved in 95% ethanol, 5°34.288'S, 134°48.416'E, Wasir, northeast Aru, 1–5 m, clove oil & hand net, field number MVE-16-077, 6 Dec 2016, M.V. Erdmann.

#### Diagnosis.

A species of *Eviota* with a cephalic sensory-canal pore pattern lacking only the IT pore, pectoral-fin rays not branched, dorsal/anal-fin formula 9/8, 5^th^ pelvic-fin ray 0–16% of 4^th^ ray; dark triangular spot on area of preural centrum followed by a narrow dark vertical line over end of hypural plate; caudal fin crossed by six dark vertical bars, naris long and black, body slender 17–20% SL; snout long, 4–6% SL, and usually 16 pectoral-fin rays.

#### Description.

Dorsal-fin elements VI+I,9, first dorsal triangular in shape, second spine slightly elongated in males, all second dorsal-fin soft rays branched, last ray branched to base; anal-fin elements I, 8, all soft rays branched, last ray branched to base; pectoral-fin rays 16 (15[1], 16[5], 17[1]), all unbranched, pointed, reaching to below second dorsal fin; 5^th^ pelvic-fin ray variable ~ 8% (0–16) of length of 4^th^ pelvic-fin ray; 4^th^ ray with 6 branches, 4 segments between consecutive branches of 4^th^ pelvic-fin ray, pelvic-fin membrane well developed, no basal membrane; caudal fin with 11 branched and 17 segmented rays; lateral-line scales 24; transverse scale rows 7; urogenital papilla of male smooth, long and narrow, expanded into a lateral horn at tip; female papilla smooth, bulbous, with short finger-like projections on end; front of head rounded at an angle of ~ 60° from horizontal axis; mouth slanted obliquely upwards, forming an angle of ~ 60° to horizontal axis of body, lower jaw not projecting; maxilla extending posteriorly to front of pupil; anterior tubular nares black, extending past rear margin of upper lip; gill opening extending forward to below posteroventral edge of preoperculum; cephalic sensory-pore system missing only IT pore, cutaneous sensory papilla system similar to papilla pattern B-1 (of Lachner and Karnella 1980).

#### Measurements.

In percent SL, value of holotype followed by range and mean of holotype and six other paratypes in parentheses. Head length 28 (28–32, 30); origin of first dorsal fin 36 (32–36, 34); origin of second dorsal fin 55 (52–58, 55); origin of anal fin 62 (54–62, 59); caudal-peduncle length 26 (23–31, 28); caudal-peduncle depth 13 (13–15, 14); body depth 20 (17–20, 19); eye diameter 9 (8–10, 9); snout length 6 (4–6, 5); upper-jaw length 9 (females 7–9, 8; males 9v12, 10.0); pectoral-fin length 37 (29–41, 34); greatest pelvic-fin length 36 (30–38, 33).

#### Color in life.

(Figure 6). Background color of head and body translucent gray. Body with black subcutaneous lateral stripe, stripe beginning over abdomen as a large irregular blotch, narrowing posteriorly and terminating over caudal-fin base, stripe interrupted along dorsal edge by a series of 6 or 7 short and narrow white dashes. Black blotch over abdomen with several distinct iridescent yellow-white spots over dark area. Ventral portion of flank with six evenly-spaced dark spots, starting above origin of anal and continuing onto caudal peduncle, each spot loosely connected by faint dark pigment to aforementioned black lateral stripe, and each separated from one another by small yellow-white iridescent spots. On dorsal portion of flank, anterior margins of scales strongly marked with melanophores, dark scale margins coalescing along dorsal midline to loosely form a series of 14 black spots extending from predorsal area to caudal peduncle. A bold black spot at caudal-fin base consisting of two parts, a posterior narrow line centered over posterior end of hypural plate, the anterior half a roundish spot that looks triangular in shape with one point against the line when preserved. Yellow spots dorsal and ventral dark marking on the caudal peduncle, positioned above and below the posterior end of the triangular blotch and the vertical line. Reddish color of gill filaments shows through cheek from eye to pectoral-fin base. Pectoral-fin base crossed posterioventrally by a white line extending onto rays, connecting to white spot above base. A narrow red line below eye at 6 o’clock position extending down across end of jaws; this color feature is usually prominent but can also be muted quickly. Snout bright white, top of head white, peppered with melanophores, a reddish horseshoe-shaped mark behind each eye with the opening towards the eye. Jaws, underside of head and belly translucent gray. Two red stripes at front of eye, one at 9 o’clock position the other at 10 o’clock, running forward to meet at anterior tubular naris to form a V. Eye with black pupil surrounded by a narrow gold ring, iris pinkish and crossed by reddish horizontal stripe at center of pupil, a similar stripe on iris below pupil. Upper half of iris reddish with irregular pink marks. All fin rays and spines with a reddish tinge, a distinct reddish area at caudal-fin base.

#### Color in preservative.

(Figure 7). Background color of head and body light yellow. Scale anterior margins outlined with melanophores, a narrow line of melanophores laterally over length of vertebral column. A bold black spot at caudal-fin base consisting of two parts, a posterior narrow line centered over posterior end of hypural plate, the anterior half triangular in shape with one point against the line. A series of 11 black spots extending along dorsal-fin bases onto caudal peduncle. Six postanal spots, two above anal fin. Side of head with a band of melanophores under eye at 6–8 o’clock positions extending down across jaws. Another dark band behind eye at 2 o’clock position above preoperculum. Side of head peppered with melanophores. Pectoral-fin base crossed by a narrow posteroventral line of melanophores. Top of head and nape crossed by four bands of melanophores, the first behind the eyes, the last at the first dorsal-fin base, each band with a central horizontal black line. First dorsal fin with narrow band of melanophores along its lower quarter, distal end of spines with melanophores. Second dorsal fin similar to first but basal dark band ~ 1/3–1/2 of fin and distal margin with dark band. Anal fin mostly black except at its base and distal margin. Pectoral and pelvic fins immaculate. Caudal fin crossed by six or seven vertical bands of melanophores with peppering at dorsal and ventral portions of fin.

#### Etymology.

The specific epithet is an adjective derived from the Latin *longus* (long) and *rostrum* (snout), alluding to the relatively long snout of this species compared to others in the complex.

#### Distribution and habitat.

Currently known only from the southern coastal region of West Papua, from southern Raja Ampat (based on photos only) to Fakfak, Kaimana, and the Aru Archipelago, although possibly more widespread including perhaps to Australia. Observed in shallow depths of 2–8 m on coastal reefs exposed to significant terrigenous influences (freshwater influx and sedimentation) and moderate to strong currents. Observed individually on coralline algae and dead coral substrates.

### Eviota marerubrum

Taxon classificationAnimaliaPerciformesGobiidae

Tornabene, Greenfield & Erdmann, 2021

76758D14-5D72-5D5C-85E2-C86600E1AD46

**Figure 8.**
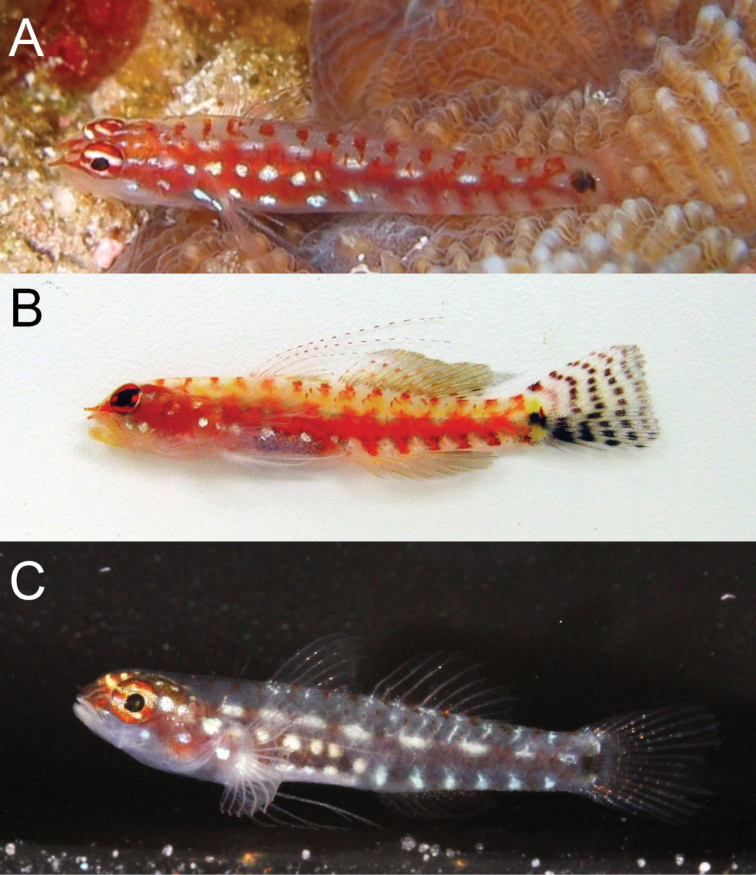
*Eviotamarerubrum*, live. **A, B** Egypt (photograph J. Herler, with permission) **C** Israel (photograph R. Holzman, with permission).

**Figure 9.**
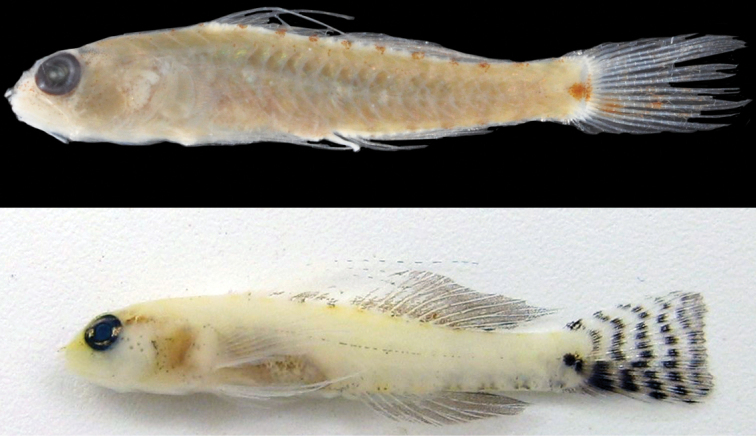
*Eviotamarerubrum*, preserved **A** holotype, USNM 218035 **B** freshly preserved specimen from Egypt, 16.5 mm SL (photograph J. Herler, with permission).

Red Sea Dwarfgoby Figures 8 , 9

Eviota cf. zebrina . Greenfield et al. (2019: 64, fig. 9 (Red Sea).
Eviota zebrina (non Lachner & Karnella). Atta et al. (2019: 8); Troyer et al. (2018: 8); Meadows et al. (2014: 3, figs 2, 3); Golani and Bogorodsky (2010: 47); Herler (2007: 85); Herler and Hilgers (2005: 114, fig. 8).

#### Material.

***Holotype*.** USNM 218035, 14.1 mm SL male, Bay at El Himeira, Egypt, Gulf of Aqaba, Red Sea, 21–27 m, 9 September 1969, V. Springer et al. ***Paratypes*.** USNM 447872 (collected with holotype), 105, Bay at El Himeira, Egypt, Gulf of Aqaba, Red Sea, 21–27 m, 9 September 1969, Victor Springer et al.; USNM 218034, 163, Bay at El Himeira, Egypt, Gulf of Aqaba, Red Sea, 9–12 m, 8 September 1969, V. Springer, G. Raz & L. Hughes-Gannes; UW 159939 (previously in USNM 218034), 2, Bay at El Himeira, Egypt, Gulf of Aqaba, Red Sea, 9–12 m, 8 September 1969, V. Springer, G. Raz & L. Hughes-Gannes; CAS 247198 (previously in USNM 218034), 2f, Bay at El Himeira, Egypt, Gulf of Aqaba, Red Sea, 9–12 m, 8 September 1969, V. Springer, G. Raz & L. Hughes-Gannes; USNM 218031, 23, Bay at El Himeira, Egypt, Gulf of Aqaba, Red Sea, 0–18 m, 16 July 1969, V. Springer, A. Amir, G. Raz & H. Harpaz.

#### Non-type material.

CAS 239041, 1, preserved in 95% ethanol, Al Lith, Shi’b Habil reef, Station 24, exposed inner shelf, 8.1 m depth, 30 Jan 2015, D.J. Coker, J.D. DiBattista, T.H. Sinclair-Taylor, and M.L. Berumen; CAS 239042, 1, preserved in 95% ethanol, Al Lith, Marmar reef, Station 29, sheltered outer shelf, cave next to station, 10.3 m depth, 31 Jan 2015, D.J. Coker, J.D. DiBattista, T.H. Sinclair-Taylor, and M.L. Berumen; CAS 239043, 1, preserved in 95% ethanol, Thuwal, Abu Madafi reef, Station 31, exposed outer shelf, 14 m depth, 5 Feb 2015, D.J. Coker, J.D. DiBattista, T.H. Sinclair-Taylor, and M.L. Berumen. Examined by Lachner and Karnella (1978), all from the Red Sea: USNM 218030, 3 (13.0–16.5); USNM 218031, 23 (8.4–15.3); USNM 218032, 6 (8.1–11.4); USNM 218033, 6 (8.8–11.2); FMNH 83851, 2; FMNH 83850, 1; BPBM 13428, 1.

#### Diagnosis.

A species of *Eviota* with a cephalic sensory-canal pore pattern lacking only the IT pore, AITO small and opening dorsally; pectoral-fin rays not branched; dorsal/anal-fin formula 8/7; 5^th^ pelvic-fin ray 8–15% of 4^th^ ray; small dark circular spot on area of preural centrum connected to a short, narrow dark vertical line over end of hypural plate; caudal fin of freshly dead specimens crossed by five or six dark vertical bars, naris long and reddish brown; eye with two distinct white horizontal stripes, one crossing through upper margin of eye, another crossing through lower margin of eye; body depth approximately 20–25% SL; usually 15 pectoral-fin rays.

#### Description.

Fin-ray counts for dorsal, anal, and pectoral fins in the following description are based on specimens examined by Lachner and Karnella (1978) from the Red Sea. We reexamined ten specimens each from three of the lots from that study from Egypt, Northern Gulf of Aqaba, Red Sea, which were used to obtain morphometrics and to confirm meristic characters. The holotype was selected from one of those three lots (USNM 218035) and all other specimens in this lot are designated as paratypes and given a new catalog number, USNM 447872. Four specimens were removed from USNM 218034, two of which are now paratypes CAS 247198 and two are paratypes UW 159939. The remaining specimens in USNM 218034, and USNM 218031, are also designated as paratypes.

Dorsal-fin elements VI+I,8 (7[1], 8[29], 9[2]), first dorsal triangular in shape, first or second spine elongated in some specimens of both sexes, ranging from slightly elongate (extending to first ray of second dorsal fin when depressed) in some smaller males and females of all sizes, to extremely elongate (extending to beyond last ray of second dorsal-fin when depressed) in largest males, all second dorsal-fin soft rays branched, last ray branched to base; anal-fin elements I, 7 (in all specimens examined), all soft rays branched, last ray branched to base; pectoral-fin rays 15 (14[3], 15[15], 16[7]), all unbranched, pointed, reaching to below second dorsal fin; 5^th^ pelvic-fin ray variable ~ 10% (8–15%) of length of 4^th^ pelvic-fin ray; 4^th^ ray with 4–6 branches, 1–3 segments between consecutive branches of 4^th^ pelvic-fin ray, pelvic-fin membrane not well developed, no basal membrane; caudal fin with 11 branched and 17 segmented rays (caudal fin broken in holotype and some paratypes, unable to count segmented rays); lateral-line scales 23 (22[2], 23[4], 24[2], 25[1], 26[1]); transverse scale rows 8 (7[6], 8[3]); urogenital papilla of male smooth, long and narrow, expanded into two lateral horns at tip; female papilla smooth, bulbous, with short finger-like projections on end; front of head sloping at an angle of ~ 50° up from horizontal axis; mouth slanted obliquely upwards, forming an angle of ~ 30° upwards from horizontal axis of body, lower jaw not projecting; maxilla extending posteriorly to posterior margin of pupil; anterior tubular nares reddish brown in life, extending past rear margin of upper lip; gill opening extending forward to below posteroventral edge of preoperculum; cephalic sensory-pore system missing only IT pore, AITO pore small and opening dorsally, cutaneous sensory papilla system similar to papilla pattern B-1 (of Lachner and Karnella 1980).

#### Measurements.

In percent SL, value of holotype followed by range and mean of holotype and ten other paratypes in parentheses. Head length 28 (27–31, 29); origin of first dorsal fin 35 (33–38, 35); origin of second dorsal fin 53 (52–59, 55); origin of anal fin 58 (56–64, 60); caudal-peduncle length 26 (22–29, 26); caudal-peduncle depth 12 (10–13, 11); body depth 25 (20–25, 22); eye diameter 9 (9–11, 10); snout length 5 (4–5, 4); upper-jaw length 11 (females 9 or 10, 10; males 10–12, 10); pectoral-fin length 23 (21–29, 24); greatest pelvic-fin length 39 (27–39, 31).

#### Color in life.

(Figure 8). Background color of head and body translucent gray. Body with broad red stripe extending from tip of snout to caudal peduncle, stripe interrupted by eight or nine iridescent white dashes evenly spaced along dorsal margin of stripe on body, dashes beginning above the operculum and terminating on caudal peduncle.

Six short red bars extending ventrally from red lateral stripe, first bar just posterior to origin of anal fin, last bar at the posterior margin of caudal peduncle, each bar separated by a white (sometimes iridescent) space. Dorsal midline with 13 or 14 small evenly spaced red spots beginning on nape at a vertical above operculum and extending posteriorly to posterodorsal margin of caudal peduncle. Abdomen and side of body behind pectoral fin with six to eight iridescent white spots over red lateral stripe. Pectoral-fin base red with iridescent white spot in center. Pair of small dark spots at base of caudal fin, anterior spot centered just anterior to origin of caudal rays and circular in shape, posterior spot more vertically elongate, located on base of caudal rays.

Head pale gray ventrally, with red snout, nares, and nape. Eyes red with two horizontal white stripes on iris, above and below pupil. Short white stripe on head extending from behind eye, in line with upper stripe on iris, to operculum; white stripe sometimes broken into two small spots rather than continuous stripe. One or two iridescent white spots, slightly smaller than diameter of pupil, on side of head posterior to jaws. First three spines of the dorsal fin with evenly spaced red spots along entirety of spine. Second dorsal-fin rays each with two red spots evenly spaced along rays. Pectoral fins and pelvic fins white. Caudal fin pale with four faint red vertical bands.

#### Color in preservative.

(Figure 9). Background color of head and body yellowish pale. Side of body without prominent markings except for a very narrow stripe line of single melanophores along lateral midline (present only in well-preserved specimens). Dorsal margin of body with 13 small evenly spaced dark spots beginning on nape at a vertical above operculum and extending posteriorly to posterodorsal margin of caudal peduncle. Dorsal and anal fins unpigmented in type series, however recently preserved specimens may have a dark band of melanophores at the base of first dorsal fin, and second dorsal and anal fins uniformly covered with small melanophores. Pelvic fins without pigment. Caudal fin with four faint brown vertical bands, band more pronounced in freshly preserved specimens.

#### Etymology.

The specific epithet is an adjective combining the Latin *maris* (sea) and *ruber* (red) referring to the type locality, the Red Sea.

#### Distribution and habitat.

Currently known only from specimens examined from the Red Sea, ranging from the Gulf of Aqaba in the northern Red Sea to the central Red Sea off the coast of Saudi Arabia.

### Eviota oculineata

Taxon classificationAnimaliaPerciformesGobiidae

Tornabene, Greenfield & Erdmann, 2021

E004A6C2-36BB-5B6B-9DF6-AD81DBB87DE4

**Figure 10.**
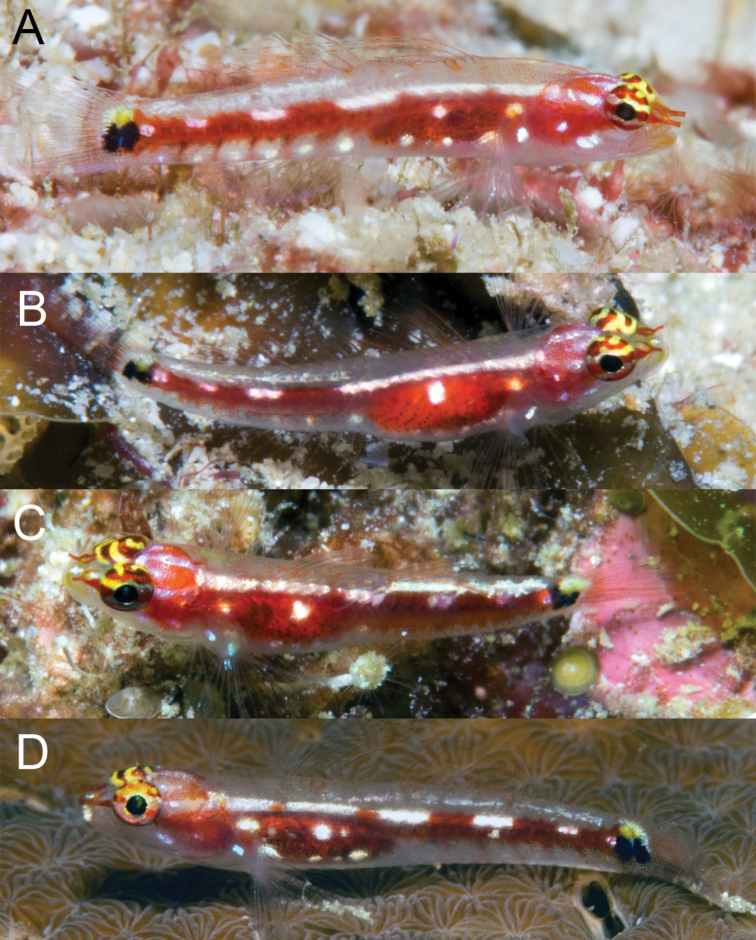
*Eviotaoculineata*, live, New Guinea.

**Figure 11.**
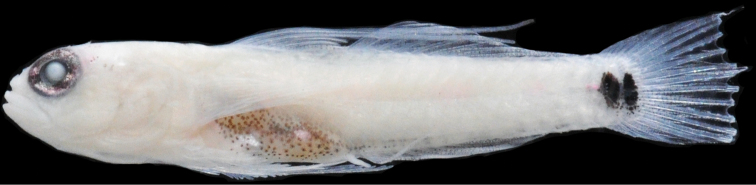
*Eviotaoculineata*, preserved holotype, CAS 247279.

False-comet Dwarfgoby Figures 10 , 11

Eviota cf. cometa Greenfield et al. 2019: 64, fig. 9 (New Guinea and Solomon Islands).
Eviota cometa (non Lachner & Karnella). Greenfield et al. (2019: fig. 8); Greenfield and Randall (2016: fig. 35); Allen and Erdmann (2012: 913); Randall (2005: 530); Suzuki et al. (2004: 131); Nakabo (2002: 1181).

#### Material.

***Holotype*.** CAS 247279, 10.9 mm SL male, tissue number COM5, preserved in 95% ethanol, 01°37.300'S, 138°43.395'E, Pulau Liki West, northern New Guinea, 30 m, clove oil & hand net, field number MVE-19-006, 12 Feb 2019, M.V. Erdmann. ***Paratypes*.** CAS 246244, 2, tissue numbers COM1 and COM2, preserved in 95% ethanol, 10°53.773'S, 150°44.637'E, Dumoulins, Milne Bay, Papua New Guinea, 20 m, clove oil & hand net, field number MVE-16-020, 23 May 2016, M.V. Erdmann. CAS 246245, 9.9 mm SL male, tissue number COM3, preserved in 95% ethanol, 09°19.954'S, 160°17.946'E, Shoal near Nugu Island, Florida Island Group, Solomon Islands, 35 m, 11 October 2016, M.V. Erdmann.

#### Non-type material.

Fiji - CAS 228684, 2, field number G02-146, Naigani Island, patch reef, clear water, coral wall overhang with black coral, 17°34.611'S, 178°40.217'E, 5.4–9. 1 m, rotenone, 4 November 2002, Greenfield, et al.; CAS 238063, 2, field number JVE-10-11-2015, Mt. Mutiny Dive site, N.E. of Viti Levu, off Nanukuloa, rubbly reef, 17°20.76'S, 178°31.35'E, 12 m, clove oil, 11 October 2015, J.V. Eyre.

#### Diagnosis.

A species of *Eviota* with a cephalic sensory-canal pore pattern lacking only the IT pore, AITO small and opening dorsally; pectoral-fin rays not branched; dorsal/anal-fin formula 8/7; 5^th^ pelvic-fin ray 8–15% of 4^th^ ray; large dark oval spot on area of preural centrum connected to a short, vertically elongate spot over end of hypural plate; caudal fin of freshly dead specimens without prominent vertical bars; naris long and reddish brown; side of body with prominent red lateral streak bordered dorsally by three to five elongate white dashes; eye with two distinct horizontal stripes, one white crossing through lower margin of pupil, one yellow crossing upper margin of pupil; body depth approximately 27–31% SL; usually 14 pectoral-fin rays.

#### Description.

Dorsal-fin elements VI+I,8, first dorsal triangular in shape, first or second spine elongated in some specimens of both sexes, ranging from slightly elongate (extending to first ray of second dorsal fin when depressed) in the smallest male, to extremely elongate (extending to beyond origin of last ray of second dorsal fin when depressed, as in holotype), all second dorsal-fin soft rays branched, last ray branched to base; anal-fin elements I, 7 (all soft rays branched, last ray branched to base; pectoral-fin rays 14 (14[3], 15[1]), all unbranched, pointed, reaching to below second dorsal fin; 5^th^ pelvic-fin ray variable, ~ 10% (8–15%) of length of 4^th^ pelvic-fin ray; 4^th^ ray with four or five branches, one or two segments between consecutive branches of 4^th^ pelvic-fin ray, pelvic-fin membrane not well developed, no basal membrane; caudal fin with 11 branched and 16 segmented rays; lateral-line scales 24 (22[1], 23[1], 24[1]), scales not countable on one specimen due to damage); transverse scale rows 7; urogenital papilla of male smooth, long and narrow, expanded into two lateral horns at tip; female papilla smooth, bulbous, with short finger-like projections on end; front of head sloping at an angle of ~ 60° up from horizontal axis; mouth slanted obliquely upwards, forming an angle of ~ 35° upwards from horizontal axis of body, lower jaw not projecting; maxilla extending posteriorly to center of pupil; anterior tubular nares extending past rear margin of upper lip; gill opening extending forward to below posteroventral edge of preoperculum; cephalic sensory-pore system missing only IT pore, AITO pore small and opening dorsally; cutaneous sensory papilla system similar to papilla pattern B-1 (of Lachner and Karnella 1980).

#### Measurements.

In percent SL, value of holotype followed by range and mean of holotype and ten other paratypes in parentheses. Head length 27 (27–31, 28); origin of first dorsal fin 37 (35–37, 36); origin of second dorsal fin 57 (54–58, 56); origin of anal fin 59 (59–62, 60); caudal-peduncle length 25 (20–29, 25); caudal-peduncle depth 11 (11–13, 12); body depth 18 (18 or 19, 18); eye diameter 9 (8–10, 9); snout length 3 (3–6, 4); upper-jaw length 8 (females 9 or 10, 9; males 8 or 9, 9); pectoral-fin length 25 (22–29, 25); greatest pelvic-fin length 30 (28–33, 31).

#### Color in life.

(Figure 10). Background color of head and body translucent gray. Body with broad red stripe extending from tip of snout to caudal peduncle, stripe interrupted by five iridescent white dashes evenly spaced along dorsal margin of stripe on body, dashes beginning above the operculum and terminating on caudal peduncle, with anterior three dashes sometimes merged as a continuous white stripe over abdomen.

Occasionally five short red bars extending ventrally from red lateral stripe, first bar just posterior to origin of anal fin, last bar at the posterior margin of caudal peduncle, each bar separated by a white (sometimes iridescent) space. Abdomen and side of body behind pectoral fin with two (sometimes three) iridescent white spots over red lateral stripe, first spot immediately above and posterior to axis of pectoral fin. Pectoral-fin base red with iridescent white spot in center. Pair of vertically elongate, broadly joined dark spots at base of caudal fin, anterior spot centered just anterior to origin of caudal rays, posterior spot located on base of caudal rays. Small yellow spot on base of caudal fin just dorsal to pair of dark spots.

Head pale gray ventrally, with red snout, nares, and nape. Eyes red with two horizontal stripes on iris, one yellow and passing through upper margin of pupil, one white and passing through lower margin pupil, dorsal margin of eye above stripe with yellow spots or mottling. Short white stripe on head behind eye, in line with upper stripe on iris, extending posteriorly to operculum; white stripe sometimes broken into two small spots rather than continuous stripe. One or two iridescent white spots, slightly smaller than diameter of pupil, on side of head posterior to jaws. First three spines of the dorsal fin with evenly spaced red spots along entirety of spine. Second dorsal-fin rays faintly tinged with red. Pectoral fins and pelvic fins white. Caudal fin lacking prominent vertical bands, lower half of caudal fin with faint red hue.

#### Color in preservative.

(Figure 11). Based on holotype, preserved in 95% ethanol. Background color of head and body yellowish pale. Head without pigment except for a faint scattering of melanophores on nape. Abdomen uniformly covered with dark melanophores, remaining side of body lacking any pigmentation except for a row of scattered melanophores along the ventral midline of body, beginning at anal fin origin on continuing to middle of caudal peduncle. Pair of vertically elongate, broadly joined dark spots at base of caudal fin, anterior spot centered just anterior to origin of caudal rays, posterior spot located on base of caudal rays. Dorsal and anal fins with light scattering of chromatophores on inter-radial membranes and on rays of posterior half of fins. Caudal fin lacking pigmentation except for a faint horizontal streak of melanophores on five rays, the first of which is just below the lateral midline of the caudal fin, followed by the four subsequent ventral rays. Pectoral fin, pectoral-fin base, and pelvic fins without pigment.

#### Etymology.

The specific epithet is an adjective combining the Latin *oculi* (eye) and *linea* (line, stripe) referring to the stripes through the eye, which distinguish this species from *E.cometa*.

#### Distribution.

**Figure 12.**
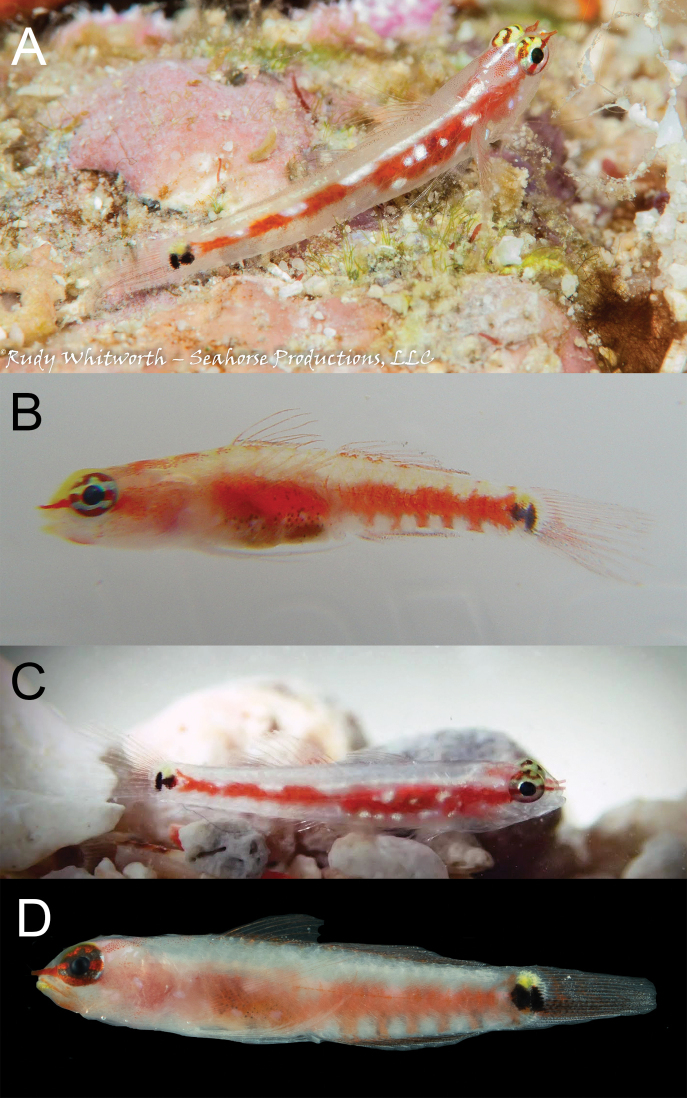
*Eviotaoculineata* photographs from non-type specimens **A** Fiji (photograph R. Whitworth, with permission) **B** Fiji **C** Fiji (photograph J. Eyre, with permission) **D** Australia (photograph C. Goatley, with permission).

Currently known only from New Guinea and the Solomon Islands, but likely occurs in Fiji and the Banda Sea, Indonesia, and the Great Barrier Reef, Australia, based on live photographs as well as specimens previously identified as *E.cometa* that possess 8/7 counts in the dorsal/anal fins (Figure 12). The species seems to prefer outer reef slopes exposed to clear oceanic water in depths of 20–35 m and is frequently observed resting individually on coralline algae outcrops or live plate corals.

#### Remarks.

**Figure 13.**
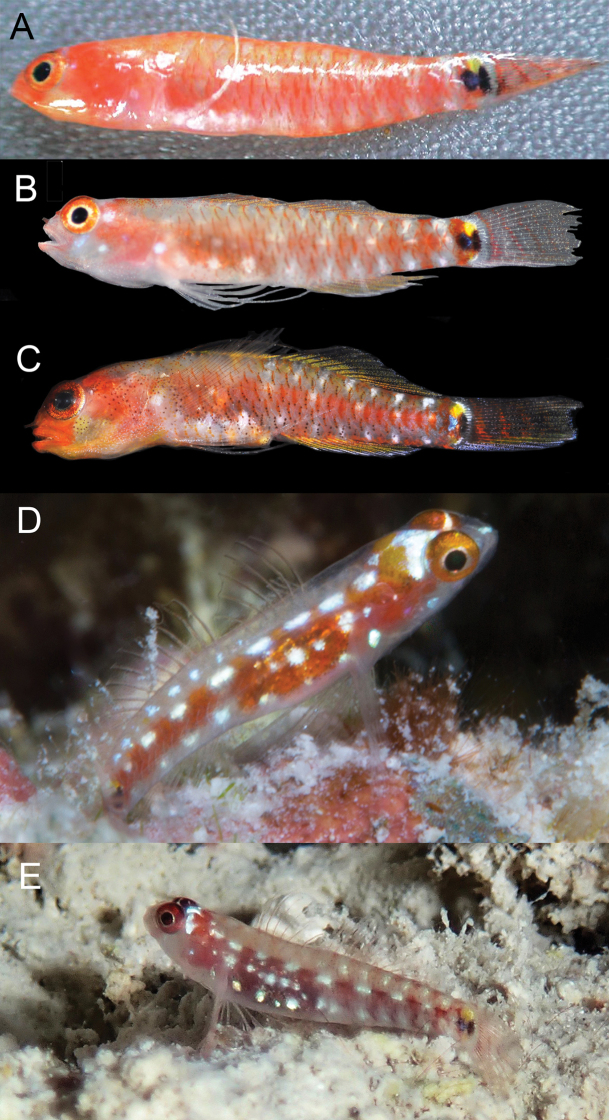
*Eviotacometa***A** freshly dead specimen from Fiji **B, C** f reshly dead specimens from Vava’u, Kingdom of Tonga (photographs M. Gómez-Buckley, with permission) **D, E** live, Fiji (photograph **E** J. Eyre, with permission).

When *E.cometa* was described, it was based on preserved material with no information on live coloration, with the holotype from Fiji. Specimens in the type series possessed both 8/7 and 9/8 dorsal/anal-fin formulas (Lachner and Karnella 1983). When Greenfield and Randall (2016) reviewed the dwarfgobies of Fiji they based their identification of preserved specimens as *E.cometa* on the description of the species by Lachner and Karnella (1983) and the underwater photographs identified as *E.cometa* in Suzuki et al. (2004), Randall (2005), and Allen and Erdmann (2012). In their review they provided two photographs (fig. 34 and fig. 35 of Greenfield and Randall 2016), one an underwater photograph by R. Whitworth taken in Fiji (Figure 12A) and a fresh specimen from CAS 222731 (Figure 13A). The underwater photograph (Figure 12A) was similar to the earlier underwater photos, showing only a clear caudal fin that was not crossed by oblique red bars, and eyes with white/yellow stripes, whereas the fin of the other fresh specimen (Figure 13A) had distinct red bars on the caudal (however, when preserved the red bars were not visible as dark bars as they are in *E.pseudozebrina*) and a solid red eye. This supported the idea that there were two species in Fiji, the type locality for *E.cometa*.

In 2017 MVE collected and photographed specimens (Figure 13D) from a silty lagoon in the Lau Archipelago, Fiji, that resembled the freshly dead specimen from Greenfield and Randall (2016, fig. 34) in that the eye was all red, different from the striped eyes in previous underwater photographs (e.g., Figures 10, 12; now *E.oculineata*). Similarly, photographs of specimens from Tonga of fish with red bars across the caudal fin and solid red eyes also appeared to be this same species (Figures 13B, C), and this was confirmed with DNA (Figure 2). Additionally, in 2020 Janet Eyre took an underwater photograph of a fish in Fiji with the same solid red eye color and red bars across the caudal fin (Figure 13E). These specimens with the red caudal-fin bars and solid red eyes have a dorsal/anal-fin formula of 9/8, the same as the holotype of *E.cometa*, whereas those individuals lacking the red bars and having striped eyes have a dorsal/anal-fin formula of 8/7 and are described here as *E.oculineata*.

The large collection of 30 *E.cometa* from Fiji, CAS 222731, came from a habitat of dead coral and silty sand, and the photographs taken by MVE (Figure 13D) and Janet Eyre (Figure 13E) were also both from protected, silty, patch reef habitat contrasting strongly with the clearer water, outer reef habitat where *E.oculineata* is found. Given the general preference of underwater photographers for clear water and healthy reefs, it is perhaps not surprising that the majority of underwater photographs in the literature appear to be *E.oculineata* and not the true *E.cometa*, given the latter species’ apparent proclivity for silty inshore reefs. Additional photographs of fishes originally identified as *E.cometa* from Lizard Island, Australia (Figure 12D) also appear to be *E.oculineata*, based on dorsal/anal fin-ray counts and coloration.

## Comparisons within the *Eviotazebrina* species complex

With the addition of the four species described here, the *E.zebrina* complex contains eight species: *E.cometa*, *E.gunawanae*, *E.longirostris*, *E.marerubrum*, *E.oculineata*, *E.pseudozebrina*, *E.tetha*, and *E.zebrina*. A combination of meristic features (e.g., counts of rays in the pectoral, dorsal, and anal fins), morphometric features (snout length, body depth), head pore patterns, and live and preserved coloration differentiate the species (Table 1). These characters are discussed in detail and are also presented in a taxonomic key below. We

**Table 1.** Diagnostic characters for distinguishing species in the *Eviotazebrina* species complex. Counts for *E.marerubrum* and *E.zebrina* include data for specimens from the Red Sea and Seychelles, respectively, listed in Table 2 of Lachner and Karnella (1978), and morphometrics for *E.zebrina* are from 15 paratypes from CAS 40599. Data for *E.cometa* are from the holotype, tissue vouchers from the phylogeny, and Fiji specimens with 9/8 D2/A rays. Measurements are given in % SL.

	* E. longirostris *	* E. zebrina *	* E. pseudozebrina *	* E. cometa *	* E. oculineata *	* E. marerubrum *	* E. tetha *	* E. gunawanae *
Type locality	Indonesia	Seychelles	Fiji	Fiji	Papua New Guinea	Egypt	Indonesia	Indonesia
Pectoral rays - mode (range)	16 (15–17)	16 (15–17)	15 (14–16)	16 (16–17)	14 (14–15)	15 (14–16)	14 (14–15)	16 (16)
D2/A rays - mode	9/8	9/8	9/8	9/8	8/7	8/7	8/7	8/7
Length 5^th^ pelvic-fin ray	absent to 15.8% of 4^th^	6–14% of 4^th^	6–16% of 4^th^	~ 10% of 4^th^	8–15% of 4^th^	8–15% of 4^th^	absent or rudimentary	~ 10% of 4^th^
AITO pore	small, opens dorsally	small, opens dorsally	small, opens dorsally	small, opens dorsally	small, opens dorsally	small, opens dorsally	large, opens anteriorly	large, opens anteriorly
IT pore	absent	absent	absent	absent	absent	absent	absent	absent
NA pores	present	present	present	present	present	present	absent	absent
Body depth - average (range)	18.7 (17.4–19.9)	19.2 (17.1–21.2)	23.2 (21.6–25.7)	21.1 (17.6–23.6)	28.4 (27.2–30.6)	22.3 (20.3–24.8)	19.6 (17.7–21.4)	19.6 (18.2–21.4)
Snout length - average (range)	5.3 (4.3–5.7)	3.4 (2.8–4.1)	4.3 (3.7–5.4)	3.7 (2.5–5.1)	4.3 (3.3–6.2)	4.3 (3.4–5.2)	4.5 (3.9–6.2)	4.4 (3.7–5.0)

**Figure 14.**
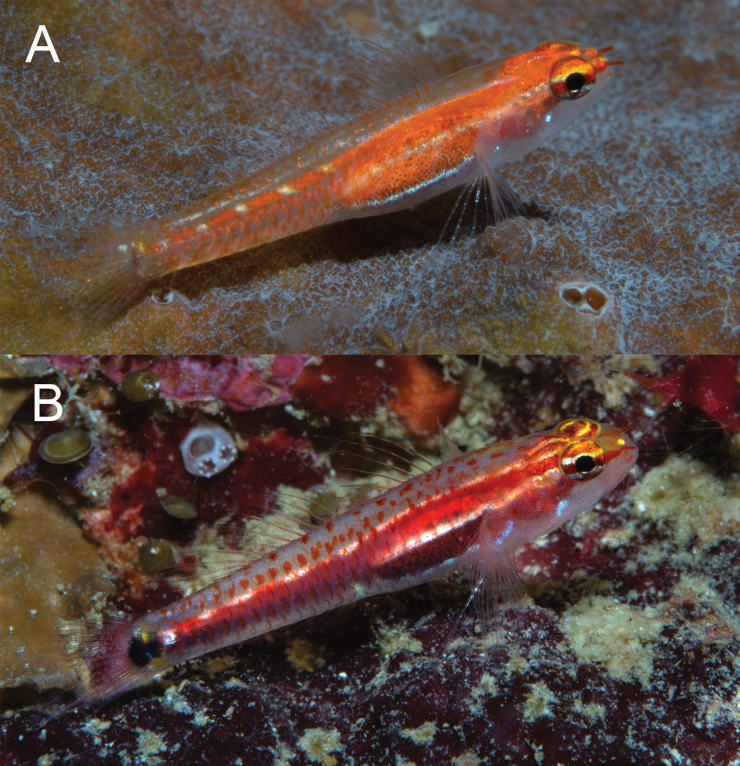
**A***E.tetha*, Cendrawasih, West Papua **B***Eviotagunawanae*, Fakfak, West Papua.

*Body coloration comparisons*. All species in this group with the exception of *E.tetha* (Figure 14A) have large, prominent dark spots at the base of the caudal fin, which are often margined dorsally (and sometimes ventrally) by smaller yellow spots in life. The side of the body of most species in this group is characterized by a prominent red streak that ends at the base of the caudal fin; however *E.longirostris* (Figure 6) and *E.pseudozebrina* (Figure 3) lack red pigment and, instead, pigment on the side of the body is predominantly black or brown. *Eviotapseudozebrina* is the least colorful of all the species in the complex, with a translucent gray background and markings that are white, brown, or black. The upper half of the eye surface is cream colored with scattered melanophores, lower half darker. The overall coloration of *E.longirostris* is bolder with red markings on the head and eye and the spines and rays with a reddish tinge.

**Figure 15.**
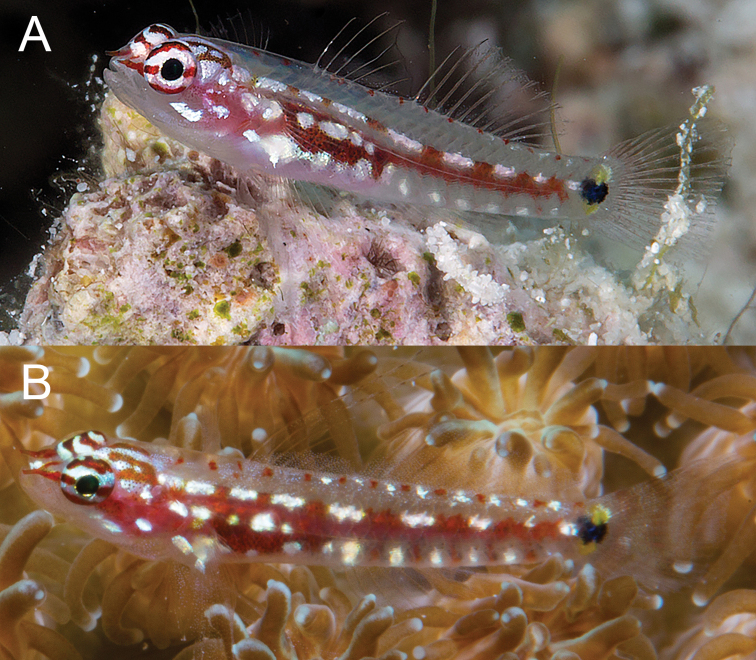
*Eviotazebrina***A** Seychelles (photograph J. Greenfield, with permission) **B** Maldives.

Five species have both a red body-stripe and the dark spot at the caudal-fin base: *E.cometa*, *E.gunawanae*, *E.marerubrum*, *E.oculineata*, and *E.zebrina*. *Eviotagunawanae* differs from the other four in having a solid white stripe on the mid-abdomen immediately posterior to the pectoral fin (Figure 14B), versus having a series of distinct white spots in this area (Figures 8, 10, 12, 13, 15). Above this region on the abdomen is a series of white stripes or dashes that extend from above the pectoral fin to the caudal fin base in all species in this complex. This series is made up of 3–6 elongate horizontal white dashes in *E.oculineata* (Figures 10, 12) versus being made up by eight or more short white dashes or spots in *E.marerubrum* (Figure 7), *E.cometa* (Figure 13), and *E.zebrina* (Figure 15). *Eviotacometa* differs from *E.marerubrum*, *E.oculineata*, and *E.zebrina* in having a large, prominent V-shaped white patch on the nape immediately posterior to the eyes (Figure 13D, E), whereas the other four species possess short horizontal white stripes in this region (Figures 8, 10, 12, 15).

**Figure 16.**
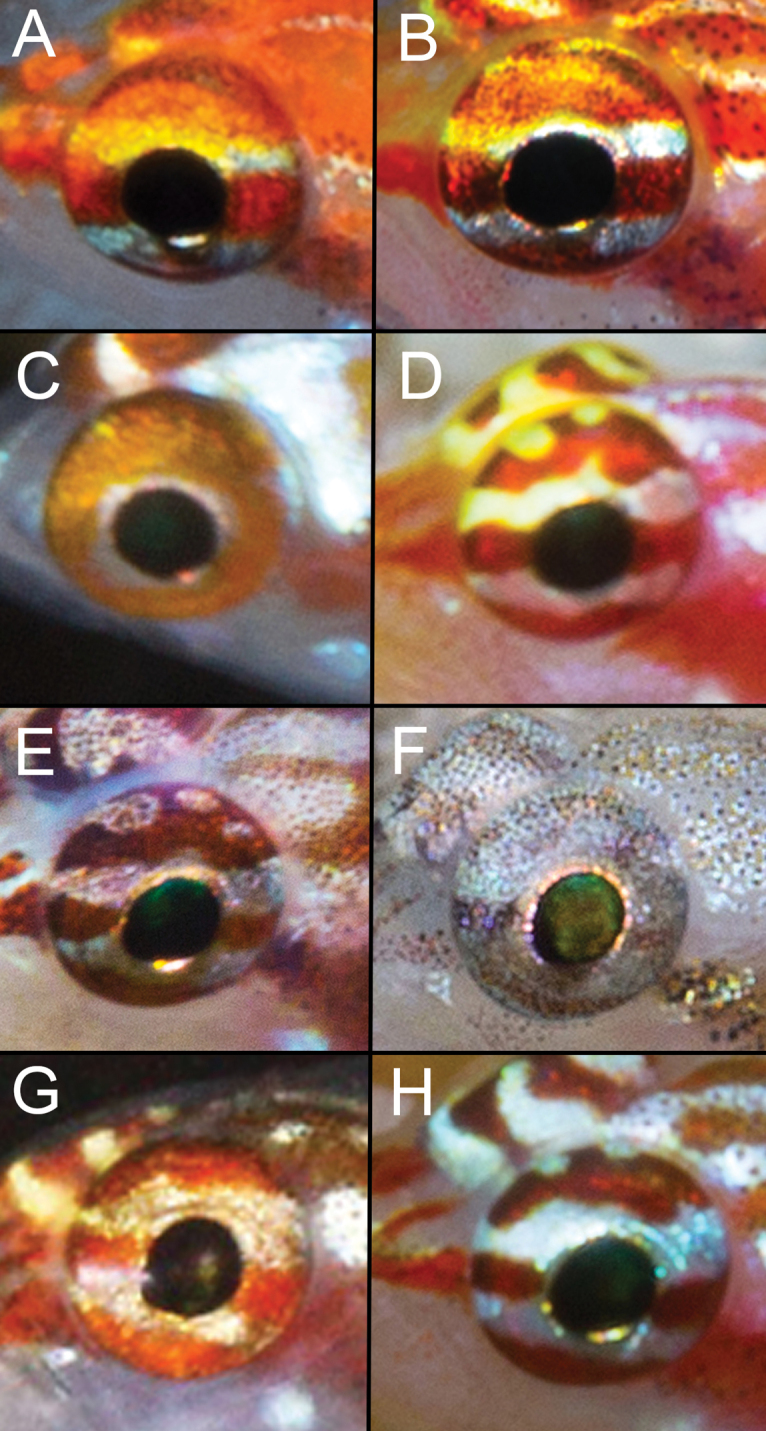
Eye coloration of the *Eviotazebrina* complex species **A***E.tetha*, Kwatisore, West Papua; **B***gunawanae*, Karas, West Papua **C***E.cometa*, Lau Archipelago, Fiji **D***E.oculineata*, Milne Bay, Papua New Guinea **E***E.longirostris*, Fakfak, West Papua **F***E.pseudozebrina*, Lau Archipelago, Fiji **G***E.marerubrum*, Israel, Red Sea **H***E.zebrina*, Laamu Atoll, Maldives (photograph **G** R. Holzman, with permission).

*Eye coloration comparisons* (Figure 16). As discussed by Greenfield (2017), eye coloration patterns in species of *Eviota* exhibit great variation and have been used in recognizing different species. Often dwelling in rock or coral crevices, dwarfgoby eyes are the body part that are most easily seen by other fishes, so it follows that eye coloration is a character that would be useful in species recognition and hence under selective pressure. Two of the species within the *E.zebrina* species complex differ from the others in lacking a reddish stripe crossing the center of the eye at the pupil. *Eviotapseudozebrina* is the most different by lacking any red in the eye, with the upper half of the eye gray and heavily peppered with melanophores, and the lower half dark (Figure 16F). From a lateral view, the entire iris of *E.cometa* is dark reddish, with the pupil surrounded by a white ring (Figure 16C). The dorsal side of the eye next to the interorbital area has a short white bar not visible laterally.

*Eviotatetha* (Figure 16A) and *E.gunawanae* (Figure 16B) both have a yellow bar crossing the eye above the pupil, a solid unbroken bar above that, a narrow white bar crossing below the pupil and a solid bar below that. *Eviotatetha* differs from *E.gunawanae* by having the bars red-orange, whereas they are darker, almost brown in *E.gunawanae*.

The remaining four species have a light bar crossing above and below the pupil, but from a lateral view only *E.marerubrum* (Figure 16G) has a solid red bar across the top of the eye, whereas the others have the upper bar broken by irregular light areas. The light bar above the pupil in *Eviotaoculineata* (Figure 16D) is yellow, unlike the other two species where the bar is white. *Eviotalongirostris* (Figure 16E) and *E.zebrina* (Figure 16) are similar but the bars in *E.zebrina* are bright red whereas they are a dark reddish color in *E.longirostris*.

**Figure 17.**
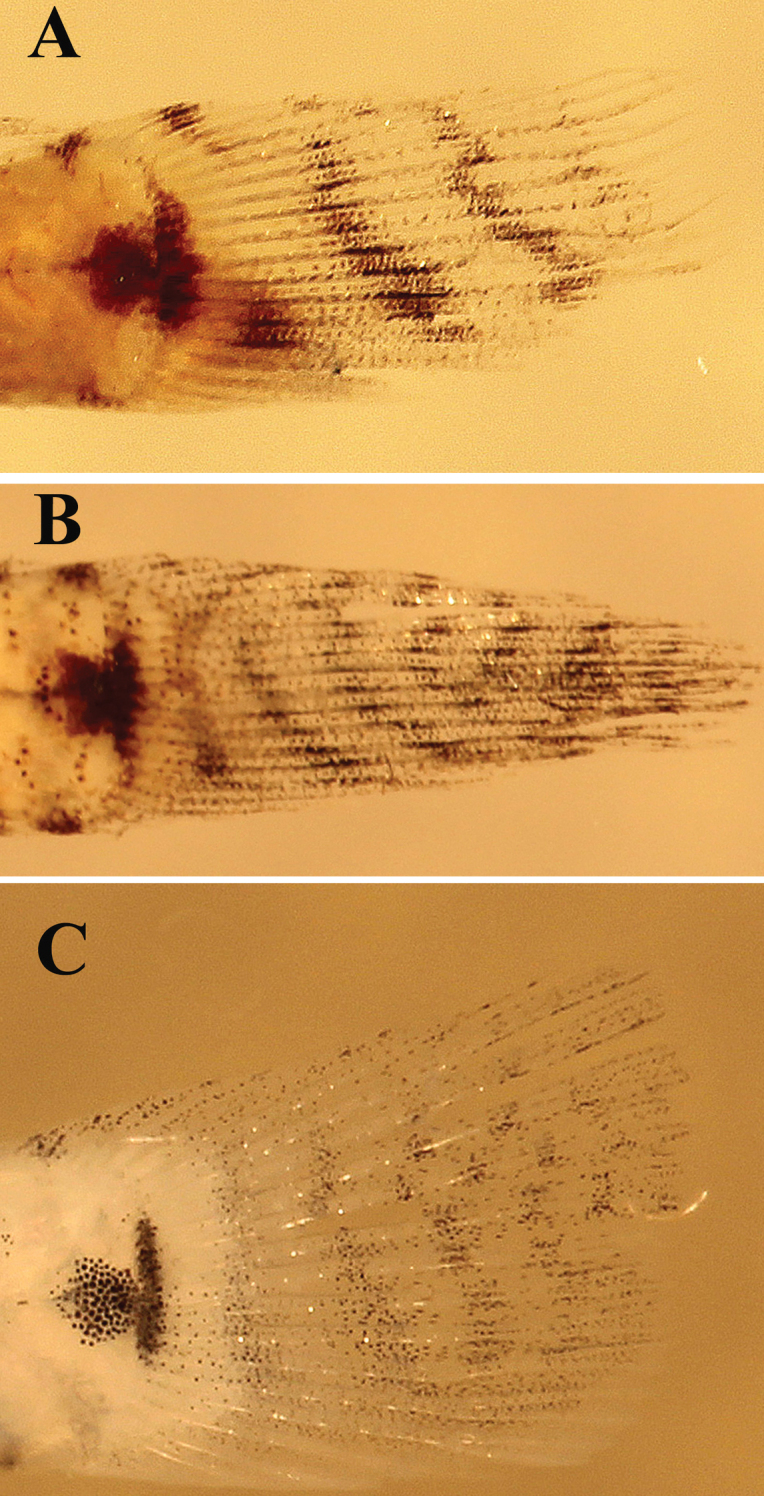
Comparison of caudal fin pigmentation in preserved specimens **A***Eviotazebrina* paratype, CAS 040599 **B***E.pseudozebrina* holotype, CAS 228614 **C***E.longirostris*, paratype, CAS 246538.

*Caudal fin markings* (Figure 17). Several species have oblique dark bars crossing the caudal fin that are obvious in freshly dead or preserved specimens, but are faint or absent in underwater photographs of live fishes. *Eviotazebrina* from the Seychelles Islands has three or four distinctive oblique black bars crossing the caudal fin. There also is a dark mark extending out from the caudal spot over the hypural plate onto the lower portion of the fin. *Eviotapseudozebrina* has 7 black bars crossing the caudal fin and no dark spot at the lower part of the fin. *Eviotalongirostris* has six or seven black bars crossing the caudal fin and no dark spot at the lower part of fin. *Eviotamarerubrum* has four black bars crossing the fin with the lower portion of the bars enlarged forming spots. *Eviotacometa* has four or five red bars crossing the fin when fresh, but these are lost in preservative. *Eviotaoculineata*, *E.tetha*, and *E.gunawanae* lack black bars on the caudal fin.

**Figure 18.**
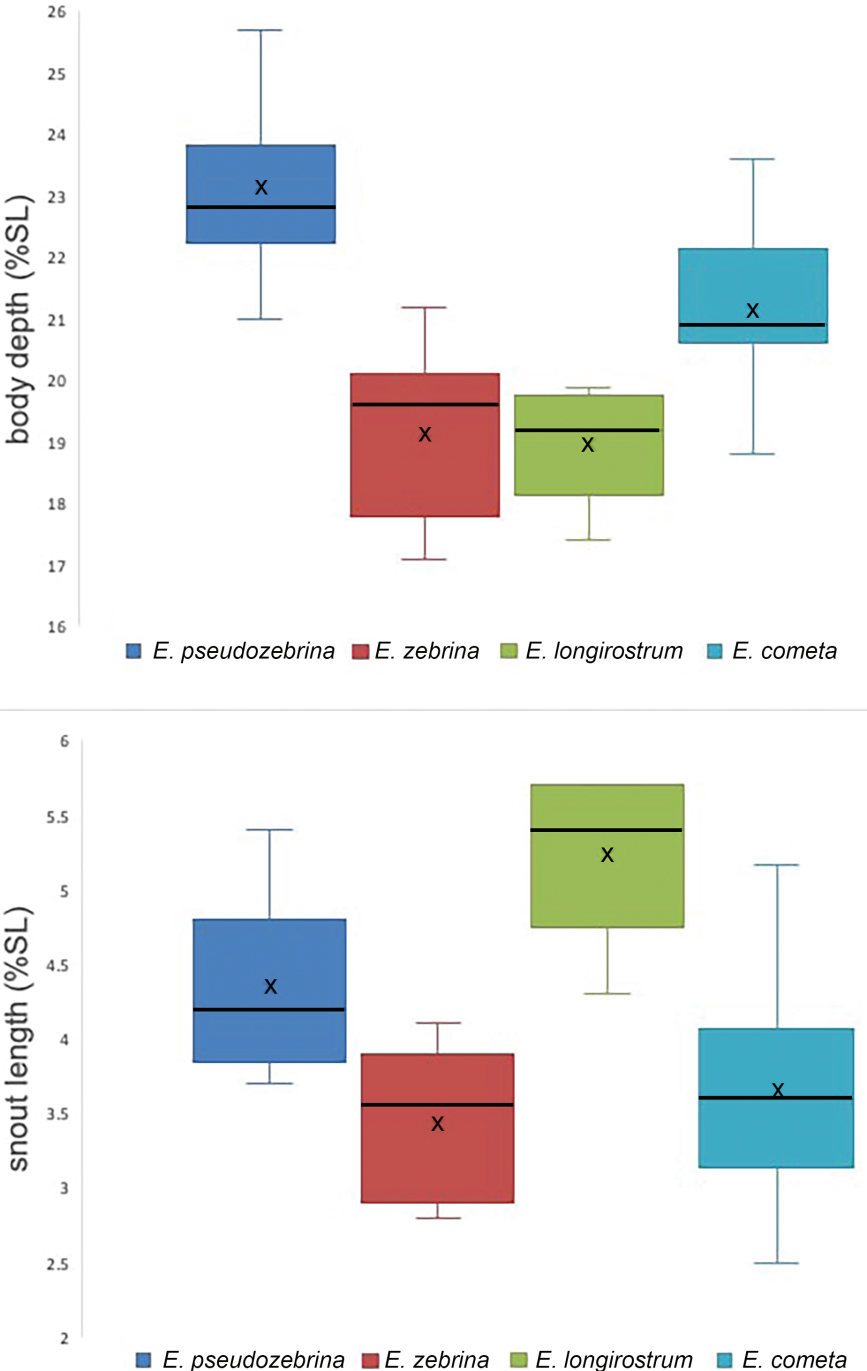
Morphometric ranges for body depth (top) and snout length (bottom) for the species of the *Eviotazebrina* complex with 9/8 dorsal-anal-fin formulas. X’s are means, bars through boxes are medians.

*Morphological characters*. *Eviotatetha* and *E.gunawanae* are similar in morphology and both can be distinguished from the rest of the complex in lacking NA pores and in having the AITO pore enlarged and opening anteriorly, versus having a small AITO pore that opens dorsally. The remaining species in the complex have all pores present except the IT pore. The six species within the complex that lack only IT pores can be distinguished primarily on the basis of dorsal/anal-fin formula and coloration. Both *E.oculineata* and *E.marerubrum* have 8/7 dorsal-anal-fin formulas (as do *E.tetha* and *E.gunawanae*) and usually have fewer pectoral-fin rays (modally 14 or 15) whereas *E.cometa*, *E.longirostris*, *E.pseudozebrina*, and *E.zebrina* have 9/8 dorsal/anal-fin formulas and modally 15 (*E.pseudozebrina*) or 16 pectoral-fin rays (*E.cometa*, *E.zebrina*, *E.longirostris*). The species in the complex with 9/8 dorsal/anal-fin formulas vary interspecifically in some morphometric features. Specifically, snout length and body depth may be useful for distinguishing between *E.cometa*, *E.longirostris*, *E.pseudozebrina*, and *E.zebrina* (Figure 18).

### Key to the species of the *Eviotazebrina* complex

Locations in parentheses only include type localities and other locations where we are confident in their occurrence based on a combination of live photographs, genetics, and specimens examined morphologically.

**Table:** 

1	NA pores absent; AITO pore large, opening anteriorly; dorsal/anal-fin formula 8/7	**2**
–	NA pores present; AITO pore small, opening dorsally; dorsal/anal-fin formula 8/7 or 9/8	**3**
2	Pectoral-fin rays 16; 5^th^ pelvic-fin ray ~10% of 4^th^; dark spot at the base of the caudal fin is larger and extends anteriorly as a partially overlapping paired spots	***Eviotagunawanae* (West Papua)**
–	Pectoral-fin rays 14 or 15; 5^th^ pelvic-fin ray rudimentary or absent; dark spot at the base of the caudal fin is restricted to the posterior end of the hypural plate	***Eviotatetha* (West Papua)**
3	Dorsal/anal-fin formula 8/7	**4**
–	Dorsal/anal-fin formula 9/8	**5**
4	Eye with two horizontal white stripes; body depth 20–25% SL	***Eviotamarerubrum* sp. nov. (Red Sea)**
–	Eye with two horizontal stripes, the upper yellow, the lower white, with yellow mottling along dorsal surface of eye; body depth 27–31% SL	***Eviotaoculineata* sp. nov. (New Guinea, Solomon Islands, Fiji, Australia)**
5	Body markings predominantly red or reddish brown	**6**
–	Body markings predominantly dark brown to black	**7**
6	Eye without prominent horizontal stripes, only a narrow gold rim around pupil over red iris; caudal fin crossed by several red vertical bars when fresh that are lost in preservation	***E.cometa* (Fiji, Tonga)**
–	Eye with pair of horizontal stripes; caudal fin crossed by several black vertical bars that are usually retained in preservation	***Eviotazebrina* (Maldives, Seychelles)**
7	Body deep 22–26, 23% SL; anterior part of dark spot at caudal-fin base a distinct rectangular shape in advance of dark line over hypural plate; upper half of eye gray, heavily peppered with melanophores	***Eviotapseudozebrina* sp. nov. (Fiji)**
–	Body more slender, depth 17–20, 19% SL; anterior part of dark spot at caudal-fin base triangular with one point against posterior dark line; upper half of eye with irregular reddish areas with white areas peppered with melanophores	***Eviotalongirostris* sp. nov. (New Guinea)**

## Discussion

The combination of molecular data, live coloration, and re-examination of preserved specimens including type series have shown that the *Eviotazebrina* complex contains at least eight species. The integrative approach taken here was first used in this complex to recognize *E.gunawanae* as being distinct from *E.tetha* (Greenfield et al. 2019), and here we expanded our taxonomic scope within the complex to recognize *E.longirostris*, *E.marerubrum*, *E.oculineata*, and *E.pseudozebrina* as being distinct from *E.zebrina* and *E.cometa*, two species that they were previously confused when molecular data and color images of live specimens were unavailable.

**Figure 19.**
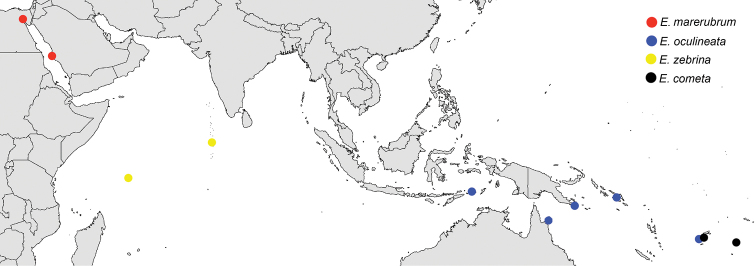
Distribution map of *E.marerubrum*, *E.oculineata*, *E.zebrina*, and *E.cometa*. Points represent localities of specimens examined in this study or verified photographs.

**Figure 20.**
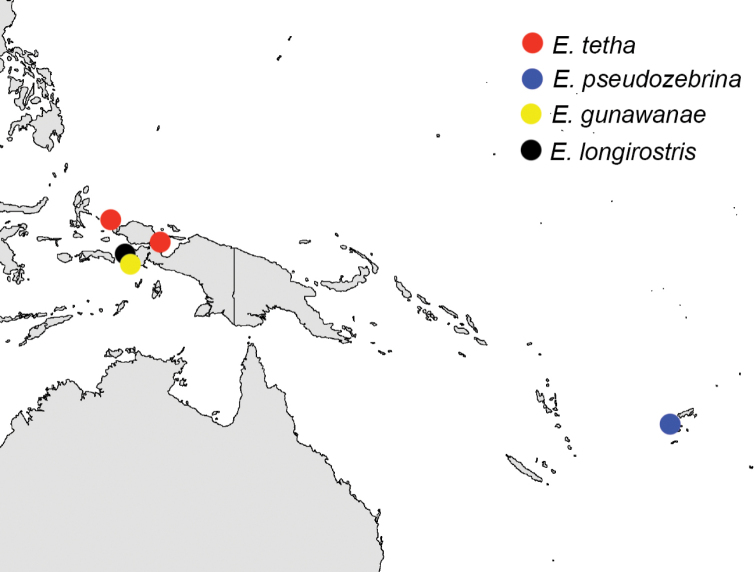
Distribution map of *E.tetha*, *E.pseudozebrina*, *E.gunawanae*, and *E.longirostris*. Points represent localities of specimens examined in this study or verified photographs.

We were unable to examine preserved specimens from throughout the range of the *E.zebrina* complex, nor would this be particularly informative given the importance of live coloration as a taxonomic character. We have also not photographed specimens or taken genetic samples throughout the *E.zebrina* complex range, so exact boundaries of species ranges given here are considered preliminary. Nevertheless, some preliminary biogeographic patterns can be observed, ranging from species pairs/groups that are predominantly allopatric to those that have ranges that overlap significantly. *Eviotazebrina* and *E.marerubrum* appear to be restricted to the Indian Ocean and Red Sea respectively, and form a clade with *E.oculineata*, which occurs from Australia and New Guinea east to Fiji, and *E.cometa* which is currently known from Fiji and Tonga (Figures 2, 19). Another large clade within this complex contains species entirely from the Central and Western Pacific (Figures 2, 20), including *E.tetha*, *E.gunawanae*, and *E.longirostris*, all from West Papua, and *E.pseudozebrina*, which is currently known only from Fiji. Interestingly, Fiji and West Papua are each home to at least three species in this complex, and are also the regions where the authors have the most live photos coupled with tissue samples. A search of museum records from 71 collections using Fishnet2 (accessed through the Fishnet2 Portal, www.fishnet2.org, 2020-11-12) showed that specimens identified as *E.cometa* or *E.zebrina* exist from many other localities including the Philippines, Australia, Thailand, China, Vietnam, Vanuatu, New Caledonia, Pohnpei, Japan, Kiribati, Palau, the Marshall Islands, Sri Lanka, and Mauritius. It is likely that increased sampling efforts in these areas will uncover additional undescribed species within this complex (and other complexes within *Eviota*) or expand the known ranges of existing species, and reveal a more complete picture of the patterns of diversification and evolution within dwarfgobies.

## Supplementary Material

XML Treatment for Eviota pseudozebrina

XML Treatment for Eviota longirostris

XML Treatment for Eviota marerubrum

XML Treatment for Eviota oculineata

## References

[B1] Akihito Sakamoto K Iwata A Ikeda Y (1993) Cephalic sensory organs of the gobioid fishes. In: Nakabo T (Ed.) Fishes of Japan with pictorial keys to the species.Tokai University Press, Tokyo, 1088–1116. [1474 pp.] [In Japanese]

[B2] Akihito Sakamoto K Ikeda Y Sugiyama K (2002) Gobioidei. In: Nakabo T (Ed.) Fishes of Japan with pictorial keys to the species.English edition. Vol. II, Tokai University Press, Tokyo, 1139–1310, 1596–1619.

[B3] Allen GE Erdmann MV (2012) Fishes of the East Indies. Vols I–III, Tropical Reef Research, Perth, 1292 pp.

[B4] Atta CJ Coker DJ Sinclair-Taylor TH DiBattista JD Kattan A Monroe AA Berumen ML (2019) Conspicuous and cryptic reef fishes from a unique and economically important region in the northern Red Sea. PLoS ONE 14(10): e0223365. 10.1371/journal.pone.0223365 PMC6822765 31671103

[B5] Fishnet2 (2020) Fishnet2. www.fishnet2.org [accessed 2020-11-12]

[B6] Golani D Bogorodsky SV (2010) The Fishes of the Red Sea – reappraisal and updated checklist. 10.11646/zootaxa.2463.1.1

[B7] Greenfield DW (2021) Addendum to the 2016 key to the dwarfgobies (Teleostei: Gobiidae: *Eviota*). Journal of the Ocean Science Foundation 38: 1–12. 10.5281/zenodo.4458248

[B8] Greenfield DW (2017) An overview of the dwarfgobies, the second most speciose coral-reef fish genus (Teleostei: Gobiidae: *Eviota*). Journal of the Ocean Science Foundation 29: 32–54. 10.5281/zenodo.1115683

[B9] Greenfield DW Erdmann MV (2021) *Eviotaflaviarma*, a new dwarfgoby from Papua New Guinea (Teleostei: Gobiidae). Journal of the Ocean Science Foundation 38: 27–34. 10.5281/zenodo.5090376

[B10] Greenfield DW Tornabene L (2014) *Eviotabrahmi* sp. nov. from Papua New Guinea, with a redescription of *Eviotanigriventris* (Teleostei: Gobiidae). Zootaxa 3793: 13–146. 10.11646/zootaxa.3793.1.6 24870157

[B11] Greenfield DW Randall JE (2016) A review of the dwarfgobies of Fiji, including descriptions of five new species (Teleostei: Gobiidae: *Eviota*). Journal of the Ocean Science Foundation 20: 25–75. 10.5281/zenodo.48268

[B12] Greenfield DW Tornabene L Erdmann MV Pada DN (2019) *Eviotagunawanae*, a new dwarfgoby from Fakfak, West Papua (Teleostei: Gobiidae). Journal of the Ocean Science Foundation 32: 57–67. 10.5281/zenodo.2616753

[B13] Herler J (2007) Microhabitats and ecomorphology of coral‐ and coral rock‐associated gobiid fish (Teleostei: Gobiidae) in the northern Red Sea. Marine Ecology 28: 82–94. 10.1111/j.1439-0485.2007.00165.x

[B14] Herler J Hilgers H (2005) A synopsis of coral and coral-rock associated gobies (Pisces: Gobiidae) from the Gulf of Aqaba, northern Red Sea. Aqua, Journal of ichthyology and aquatic biology 10: 103–132. https://aqua-aquapress.com/?p=13644

[B15] Jewett SL Lachner EA (1983) Seven new species of the Indo-Pacific genus *Eviota* (Pisces: Gobiidae). Proceedings of the Biological Society of Washington 96(4): 780–806.

[B16] Lachner EA Karnella JS (1978) Fishes of the genus *Eviota* of the Red Sea with descriptions of three new species (Teleostei: Gobiidae). Smithsonian Contributions to Zoology 286: 1–23.

[B17] Lachner EA Karnella JS (1980) Fishes of the Indo-Pacific genus *Eviota* with descriptions of eight new species (Teleostei: Gobiidae). Smithsonian Contributions to Zoology 315: 1–127.

[B18] Lanfear R Frandsen PB Wright AM Senfeld T Calcott B (2016) PartitionFinder 2: new methods for selecting partitioned models of evolution for molecular and morphological phylogenetic analyses. Molecular Biology and Evolution 34(3): 772–773. 10.1093/molbev/msw260 28013191

[B19] Meadows MG Anthes N Dangelmayer S Alwany MA Gerlach T Schulte G Sprenger D Theobald J Michiels NK (2014) Red fluorescence increases with depth in reef fishes, supporting a visual function, not UV protection. Proceedings of the Royal Society B 281: e20141211. 10.1098/rspb.2014.1211 PMC4123709 25030989

[B20] Nakabo T (2002) Fishes of Japan with pictorial key to the species, English edition II. Tokai University Press, Japan, 1749 pp.

[B21] Randall JE (2005) Reef and shore fishes of the South Pacific, New Caledonia to Tahiti and the Pitcairn Islands. University of Hawai’i Press, Honolulu, 707 pp.

[B22] Ronquist F Teslenko M van der Mark P Ayres DL Darling A Hohna S Larget B Liu L Suchard MA Huelsenbeck JP (2012) MrBayes 3.2: Efficient Bayesian Phylogenetic Inference and Model Choice Across a Large Model Space. Systematic Biology 61(3): 539–542. 10.1093/sysbio/sys029 22357727 PMC3329765

[B23] Saruwatari T JA Lopez Pietsch TW (1997) Cyanine blue: a versatile and harmless stain for specimen observations. Copeia 1997(4): 840–841. 10.2307/1447302

[B24] Suzuki T Shibukawa K Yano K Senou H (2004) . A photographic guide to the gobioid fishes of Japan. Heibosha Co., Japan, 536 pp.

[B25] Thacker CE (2003) Molecular phylogeny of the gobioid fishes (Teleostei: Perciformes: Gobioidei). Molecular Phylogenetics and Evolution 26: 354–368. 10.1016/S1055-7903(02)00361-5 12644397

[B26] Tornabene L Ahmadia GN Berumen ML Smith DJ Jompa J Pezold F (2013) Evolution of microhabitat association and morphology in a diverse group of cryptobenthic coral reef fishes (Teleostei: Gobiidae: *Eviota*). Molecular Phylogenetics and Evolution 66: 391–400. 10.1016/j.ympev.2012.10.014 23099149

[B27] Tornabene L Valdez S Erdmann MV Pezold F (2015) Support for a ‘Center of Origin’ in the Coral Triangle: cryptic diversity, recent speciation, and local endemism in a diverse lineage of reef fishes (Gobiidae: *Eviota*). Molecular Phylogenetics and Evolution 82: 200–210. 10.1016/j.ympev.2014.09.012 25300452

[B28] Tornabene L Valdez S Erdmann MV Pezold F (2016) Multi-locus sequence data reveal a new species of coral reef goby (Teleostei: Gobiidae: *Eviota*), and evidence of Pliocene vicariance across the Coral Triangle. Journal of Fish Biology 88: 1811–1834. 10.1111/jfb.12947 27021219

[B29] Troyer EM Coker DJ Berumen ML (2018) Comparison of cryptobenthic reef fish communities among microhabitats in the Red Sea. PeerJ 6: e5014. 10.7717/peerj.5014 PMC6011822 29938133

[B30] Ward RD Zemlak TS Innes BH Last PR Hebert PDN (2005) DNA barcoding Australia’s fish species. Philosophical Transactions of the Royal Society, series B 360: 1847–1857. 10.1098/rstb.2005.1716 PMC1609232 16214743

[B31] Yamada T Sugiyama T Tamaki N Kawakita A Kato M (2009) Adaptive radiation of gobies in the interstitial habitats of gravel beaches accompanied by body elongation and excessive vertebral segmentation. BMC Evolutionary Biology 9: e145. 10.1186/1471-2148-9-145 PMC2709658 19558710

